# Recent applications of machine learning and deep learning models in the prediction, diagnosis, and management of diabetes: a comprehensive review

**DOI:** 10.1186/s13098-022-00969-9

**Published:** 2022-12-27

**Authors:** Elaheh Afsaneh, Amin Sharifdini, Hadi Ghazzaghi, Mohadeseh Zarei Ghobadi

**Affiliations:** 1 Independent Researcher, Tehran, Iran; 2 Sedna Teb Mobin, Tehran, Iran

**Keywords:** Type 1 Diabetes Mellitus, Type 2 Diabetes Mellitus, Gestational Diabetes Mellitus, Machine learning, Deep learning, Blood glucose

## Abstract

Diabetes as a metabolic illness can be characterized by increased amounts of blood glucose. This abnormal increase can lead to critical detriment to the other organs such as the kidneys, eyes, heart, nerves, and blood vessels. Therefore, its prediction, prognosis, and management are essential to prevent harmful effects and also recommend more useful treatments. For these goals, machine learning algorithms have found considerable attention and have been developed successfully. This review surveys the recently proposed machine learning (ML) and deep learning (DL) models for the objectives mentioned earlier. The reported results disclose that the ML and DL algorithms are promising approaches for controlling blood glucose and diabetes. However, they should be improved and employed in large datasets to affirm their applicability.

## Introduction

Diabetes is a chronic metabolic disease that happens when the blood glucose is higher than usual. It has been predicted that the number of diabetic patients would reach about 642 million in 2040 [1]. Diabetes is categorized into three common types, namely, Type 1 Diabetes Mellitus (T1DM), Type 2 Diabetes Mellitus (T2DM), and Gestational Diabetes Mellitus (GDM). The mentioned categories are specified by dysregulation of protein metabolism, lipid, and carbohydrate [2]. In addition, there are some other particular kinds of diabetes such as maturity-onset diabetes of the young, neonatal diabetes, and also diabetes due to some diseases like the exocrine pancreas, and chemical- or drug-induced diabetes [3].

T1DM is classified as a chronic autoimmune disease that is a result of elevated blood glucose levels (hyperglycemia; glucose levels > 180 mg/dL) [4]. It causes insulin deficiency that arises from the loss of the pancreatic islet β-cells [5]. Previously, T1DM has been noticed as a disorder for children and adolescents, however, age at the symptomatic beginning has not been considered a limiting factor over the past decade [6]. T1DM is connected with the attendance of autoantibodies several years before the start of symptoms since they can be regarded as a biomarker of autoimmunity. The autoantibodies representing T1DM target insulin, zinc transporter 8 (ZNT8)6–8, or insulinoma-associated protein 2 (IA­2). T1DM as a polygenic illness is impressed by environmental agents. In addition, genetic risk factors are essential but not adequate. People with HLA genotypes of HLA­DQ and HLA­DR have an elevated risk of progressing T1DM [4, 7, 8]. Classic signs of hyperglycemia are generally comprising weight loss, polydipsia, abdominal symptoms, polyuria, ketoacidosis, and headaches. The American Diabetes Association (ADA) introduced the diagnostic criteria for diabetes mellitus in 2016 [9]. They rely on symptoms of abnormal glucose metabolism such as insulinogenic and evidence of β­cell-targeted autoimmunity, irrespective of age and diabetes type. If patients show clinical signs of T1DM without the presence of autoantibodies, the ADA diagnoses it as a group of idiopathic T1DM [4]. Furthermore, multiple environmental factors including the timing of the first introduction of food [10], viral infections [11], and gestational infections [12] can contribute to the development of T1DM.

T2DM is the most common type of diabetes so that it develops in more than 90% of all cases. The main reason for developing T2DM is the impaired secretion of insulin by pancreatic β‑cells, generally because of insulin resistance in adipose tissue 1 (BOX 1), liver, skeletal muscle, and liver [13].

Prediabetes occurs before hyperglycemia, which is a high-risk situation that predisposes subjects to the development of T2DM. Prediabetes can be determined by one of the following conditions: elevated glycated Hemoglobin A1c (HbA1c) levels, Impaired Fasting Glucose (IFG) levels, and Impaired Glucose Tolerance (IGT). Cases with IFG levels are specified by Fasting Plasma Glucose (FPG) measures greater than normal. IGT is characterized by impaired late insulin secretion after a meal and insulin resistance in muscles [14]. People with prediabetes have HbA1c amounts between 5.7 and 6.4%. The rates of annual conversion of prediabetes to T2DM are from 3 to 11% per year [2, 15].

T2DM is heritable and the probable risk of getting T2DM is higher among siblings of a T2DM patient than in families without any T2DM patient [16]. The risk of getting T2DM is higher when the mother has this disease in comparison with when the father has it. Also, the risk of T2DM is noticeably elevated with a non‑normal fasting glucose concentration of > 5.5 mmol.l^–1^ or a body mass index (BMI) of ≥ 30 [17]. The genetic studies revealed a Single‑Nucleotide Polymorphism (SNP) in TCF7L2, CDKAL1, SLC30A8, CDKN2A, FTO, HHEX, CDKN2B, GCKR, IGF2BP2, and many others in T2DM cases. However, these genetic variants only elevate the risk by 10–20% [2]. T2DM detection can be assessed based on FPG levels, increased plasma glucose test, 2‑hour post‑glucose‑load glucose level, or HbA1c. Moreover, there is a linear correlation between cardiovascular disease and glycemia with no obvious threshold. The risk of developing distal symmetric peripheral polyneuropathy and diabetic nephropathy is raised with hyperglycemia lower than those accompanied by diabetic retinopathy [18].

The US Preventive Services Task Force (USPSTF) recommended that adults higher than 45 years, people who have a first‑degree relative with diabetes, and ones who are obese or overweight should be screened in early care settings [19]. T2DM control is complex due to many pathophysiological disorders and the ‘ABCDE’ conditions (Age, Body weight, Complications, Duration, Education and Expense, and Etiology) [2, 20].

Gestational diabetes mellitus is defined as any occurrence of hyperglycemia identified in pregnancy ranging from mild IGT or IFG diagnosed in pregnancy to detecting higher glucose levels [21]. Several risk factors have been recognized for GDM including a history of gestational diabetes, a family history of T2DM, ethnicity, advanced maternal age, lifestyle factors, and diet [22]. Moreover, psychosocial and environmental factors such as endocrine disruptors, organic pollutants, and depression in the first and second trimesters have been proposed as possible risk factors for developing GDM [21]. Genetic factors can also be involved in progressing GDM, however, the data is limited and contradictory [21].

The metabolic irregularities underlying GDM comprise β-cell defects and elevated insulin resistance. These deficiencies probably exist before conception in many patients and are often quite asymptomatic. There is no unique diagnostic protocol or criteria that have been universally accepted for GDM. The International Association of Diabetes and Pregnancy Study Groups (IADPSG) published several recommendations in 2010 for the diagnosis and classification of hyperglycemia in pregnancy as follows: women with equal levels of glycemia-associated risk of detrimental pregnancy results should be grouped by the same procedure and the values of threshold blood glucose should be internationally standardized [23]. The IADPSG also recommended a ‘one-step’ approach containing an Oral Glucose Tolerance Test (OGTT) at 24–28 weeks of gestation and suggested GDM diagnostic thresholds. It relies on an adjusted odds ratio amount of 1.75 for delivering an infant impressed by critical fetal complications of maternal hyperglycemia, namely, increased cord blood C-peptide levels, elevated size at birth, and increased adiposity. The IADPSG also stated that undetected T2DM in pregnant women is dramatically prevalent in specified populations and recommended that these individuals should be diagnosed preliminarily in pregnancy and categorized as ‘overt diabetes’ [21].

Machine learning (ML) is the application of different computer algorithms that can be ameliorated spontaneously through testing and by the utilization of data. The algorithms create a model that relies on sample data, called training data, in order to predict or make decisions [24, 25]. Deep Learning (DL) is a special subcategory of ML which is a neural network with three or more layers [26]. Moreover, DL algorithms try to learn high-level secessions in data by employing hierarchical architectures. The major properties of deep learning consist of adaptability to the features and learning from the data on their own. DL causes some developments including the fast elevating chip processing capabilities, a remarkable decreasing the cost of computing hardware, and significant improvements in the ML algorithms. Particularly, ML and DL algorithms have advantageous and are applicable in diagnosing and forecasting diseases [27, 28]. They typically include performing several steps. At first, the high-throughput data containing many features are introduced to the learning process. Afterward, data is pre-processed to remove outliers and diminish the space dimensionality by excluding the disjointed data or finding the desired data. In the next step, the algorithms are developed proportional to the aim of the study. Then, the model is tested in external data to compute the performance of the developed method using some metrics such as the Receiver Operating Characteristic Curve (ROC), the Area Under the Curve (AUC), Mean Absolute Relative Difference (MARD), Root-Mean-Square Error (RMSE), Mean Squared Error (MSE), accuracy, precision, recall, F-measure, log loss, etc. Multiple ML and DL methods have been applied to various aspects of diabetes. Among them, ML approaches including Logistic Regression (LR), Extremely Gradient Boosting (XGBoost), Gradient Boosting Machine (GBM), Random Forest (RF), AdaBoost, Support Vector Machine (SVM), Least Absolute Shrinkage And Selection Operator (LASSO) regression, Bayesian Network (BN), K-Nearest Neighbor (K-NN), Artificial Neural Networks (ANNs), and ensemble algorithms as well as DL methods comprising Recurrent Neural Networks (RNNs), Long Short-Term Memory Networks (LSTMs), Gated Recurrent Unit (GRU), Convolutional Neural Networks (CNNs), and reinforcement learning (LR) have been utilized more than others. Computational algorithms have been widely employed for handling diabetes data. However, the analysis of diabetes data is complicated because most of the relevant data are nonlinear, non-normal, and correlation-structured [29]. Therefore, ML algorithms have been employed for controlling, classification, predicting, and management of diabetes.

There are several reviews that overview various machine learning and deep learning models for the classification of data-driven blood glucose patterns as well as the prediction of diabetes and hypoglycemia [30–32]. In this review, we survey the most recently developed machine learning and deep learning algorithms for various aspects of prediction, diagnosis, and management of all diabetes types in more detail. To our best knowledge, there is no such comprehensive review to overview different aspects regarding all diabetes types. We tried to cover the most recent published papers that developed and applied ML and DL methods for the following purposes: early diagnosis and prediction of diabetes; prediction of blood glucose; detection of blood glucose; Insulin resistance predicting models; determination of the start and effect of treatment; risk assessment of Diabetes; dietary and insulin dose modifications; and diabetes management. Table 1 summarizes some applied ML and DL models to construct each model. Moreover, the publicly available datasets are specified in bold.

**Table 1:** Some applied ML models in the published papers. The publicly available dataset are mentioned in bold

Sample number	ML models	Refs.
**Early diagnosis and prediction of diabetes**		
**T2DM**		
15,005 subjects with age ≥ 3	XGBoost, DNN, and RF	[63]
1512 subjects	LR, RF, Naive Bayes (NB), SVM, XGBT, ANN, K-nearest neighbor (KNN), DT, XceptionResNet 50, DenseNet121, Vgg16, Vgg19, and InceptionV3, Stacking model of non-invasive variables and the Resnet50 model	[53]
530 participants: 272 were diabetic patients and 258 were non-diabetic patients	Deep autoencoder learning algorithm with CNN networks and deep radial basis function neural network (RBFNN) classifier	[52]
217 participants with diabetes, prediabetes and normal conditions	SVM, K-nearest neighbors, RF, XGBoost, hybrid feature selection-XGBoost	[91]
2371 T1-weighted whole-body MRI data sets	DenseNet architecture	[54]
8454 subjects over five years of follow- up	XGBoost, SVM, LR, RF, and ensemble algorithms	[64]
16,429 men and non-pregnant women ≥ 20 years of age	ANN, LR, and RF models	[55]
453,487 T2DM patients	Reverse engineering and forward simulation (REFS)	[124]
82 obese women (40 non-diabetic and 42 diabetes)	Separability-correlation measure (SCM) and ANN	[57]
13,309 Canadian patients	GBM and LR	[92]
**Kaggle diabetes dataset**	RF	[58]
1492 healthy individuals	SVM	[59]
10 patients	LR, CNN, Multi-Layer Perceptrons (MLPs), and ensembling methods	[60]
4870 subjects (2955 females and 1915 males)	Bayes classifier and LR	[79]
768 individuals, 500 healthy and 268 with T2DM (**UCI Machine Learning Repository: Pima Indians diabetes data set**)	AIRS2 and MAIRS2	[61]
**Pima Indian women**	DT and LR	[65]
2970 youth aged 12–19 years (**NHANES dataset**)	LR, LogitBoost, and decision tree	[66]
746 subjects	SVM, XGBoost, RF, and their combinations	[62]
**GDM**		
22,242 singleton pregnancies (3182 women developed GDM)	RF, logistic, decision tree, XGB, GDBT, LGB, AdaBoost, Vote, logistic regression with RCS and stepwise logistic regression	[75]
490 pregnant women, 215 with GDM and 275 controls	SVM and light gradient boosting machine (lightGBM)	[76]
588,622 pregnancies from 368,351 women	Gradient-boosting machine model constructed by decision-tree base-learners	[74]
4378 cases	CSHM, BN, LR, CHAID tree, SVM, and NN	[77]
152 women	AIRS	[80]
4771 pregnant women in early gestation	Multivariate Bayesian logistic regression using Markov Chain Monte Carlo simulation algorithm	[81]
All types of Diabetes		
2001 cases with diabetes (**Kaggle dataset**)	Filter based DT-(ID3) algorithm for features selection and Hold out, K-fold, and LOSO for classification	[83]
852 454 individuals with pre-diabetes	LightGBM	[86]
1050 curves of glucose concentration of type 1 and type 2 diabetics	Double-Class AdaBoost	[87]
268 females and 500 controls	Gaussian process (GP)-based classification approach	[88]
5301 African Americans	RF	[89]
268 females and 500 controls	Fuzzy c-means (FCM)- on adaptive network-based fuzzy inference system (ANFIS)	[90]
**Pima Indian Diabetes Dataset and Biostat Diabetes Dataset**	RLEFRBS	[95]
**Prediction of blood glucose (BG)**		
**OhioT1DM dataset**: six participants with T1D between 40 and 60 years old	SVM, extended tree classifier (ETC), and random forest classifier (RFC)	[121]
IDIAB, **OhioT1DM dataset**, and **T1DMS datasets**	Fully convolutional neural network	[101]
225 T1DM patients with 315,000 h of CGM data	Linear extrapolation, NNs, last observation carried forward, ensemble methods using LSBoost and bagging, one with error-weights, and one without error-weights	[122]
Blood glucose concentration values of 180 h in diabetic patients and GCM of every 5 min	Multi-scale blood glucose prediction model (VMD-KELM-AdaBoost)	[102]
**OhioT1DM dataset**	Autoregression with ARX model, ML-based regression models, and DL models including a TCN and a vanilla LSTM Network	[104]
10 adult T1DM subjects which was generated using the UVA/Padova T1D	Multi-layer convolutional recurrent neural network (CRNN) architecture	[105]
**OhioT1DM dataset**	LSTM-based deep RNN	[107]
104 people who had experienced at least one hypoglycemia alert value during a three-day CGM session	SVM using radial basis or linear functions, RF, LR, and K-nearest neighbor	[116]
10 T1DM patients with continuous glucose monitoring system data points	A combination of AR, SVR, and ELM	[109]
10 T1DM adults studied during 12 weeks	SVM and MLP	[119]
10,000 users with more than 1 million nights of CGM data	RF	[120]
26 participants	LSTM-NN-TF-DTW model	[143]
8501 eligible participants	LASSO regression and RF	[130]
124 CGM traces collected over 10 days	Autoregressive, autoregressive moving average, and autoregressive integrated moving average (ARIMA)) and nonlinear machine-learning procedures (SVR, feed-forward neural network (fNN), regression random forest, and LSTM-NN	[110]
Six subjects suffering from T1DM aged between 23 and 52 (average 39 ± 10)	Jump Neural Network	[154]
124 people (22,804 valid nights of data) with T1D	SVR	[117]
463 people with T1DM	Linear discriminant analysis	[118]
154 observations of in-clinic aerobic exercise in 43 adults with T1DM	Decision tree and Random forest	[126]
16 children with T1DM	Extreme learning machine (ELM)-based neural network	[127]
8 patients (320 data points) and a testing set with 8 patients (269 data points)	ELM trained feed-forward neural network	[128]
24 331 adults	Bayesian scoring algorithm	[78]
Ten male subjects with T1DM	pattern classification algorithm	[125]
27,050 adult individuals with no prior diagnosis of T2DM	XGBoost, RF, Glmnet, and LightGBM	[112]
6.8 million data points	Combination of GBD and SVR	[131]
10 patients using 70 mg/dL and 54 mg/dL as thresholds according to the consensus for Level 1 and Level 2 hypoglycemia	Developed SVM	[129]
The health data associated with 18 691 ICU stays and 14 742 critical care patients (**MIMIC-III database**)	GBT	[132]
29,601 entries from 47 different patients	SVR, WNN, KNN, RFR, GPR, ANN, and RR	[134]
54 978 inpatients who had a minimum of 4 BG measurements and took a minimum of 1 U of insulin during hospitalization	RF classification, multivariable logistic regression, stochastic gradient boosting (SGB), and naive Bayes	[114]
25 T1DM patients	RF	[135]
**OhioT1DM dataset**	LR, vanilla LSTM, and BiLSTM	[137]
**Detection of blood glucose**		
12 healthy subjects	Back-propagation neural network (BPNN) and multivariate polynomial regression	[151]
540 patients with T2DM	Nonlinear and linear predictive algorithms	[152]
2787 consecutive participants	Combination of elastic network with RF, SVM, and back-propagation artificial neural network (BP-ANN) algorithms as well as LR	[155]
1772 paired data varying from 65 ~ 492 mg/dl and 80 ~ 352 mg/dl	AdaBoost	[156]
15 patients with T1DM under free-living conditions	RReliefF, RF, Gaussian, SVR	[157]
EMR of 127 patients for the first 72 h of ICU care who upon admission to the ICU had a diagnosis of type 1 (N = 8) T2DM (N = 97) or a glucose value > 150 mg/dl (N = 22)	GBT	[133]
**Insulin resistance predicting models**		
8842 Koreans participants	LR, XGBoost, random forest, and ANN	[159]
1344 samples	HOMA-IR model	[160]
2433 T2DM patients	MIL-Boost	[161]
968 patients not affected by T2DM (**FIMMG_obs dataset**)	TyG-er	[162]
315 T1DM patients	MARSplines and ANN	[163]
**Determination of the start of treatment and its effect**		
13 904 diabetes individuals	LASSO	[164]
100 virtual adult subjects	LASSO and MLR	[166]
100 virtual subjects	GBT and RF	[165]
87 patients	Reinforcement learning	[168]
The two studies had a similar design but enrolled patients who were treatment- naïve (study 1, n = 677) or receiving background metformin (study 2, n = 686)	RF and classification tree algorithms	[169]
12,147 commercially-insured adults and Medicare Advantage beneficiaries with prediabetes or diabetes	RL both with and without regularization and/or stepwise feature selection, Tree-based models, SVM, multivariate adaptive regression splines, and flexible discriminants	[170]
1270 patients with T2DM	Weighted SVM	[171]
100 virtual adults	Neural networks	[167]
3029 patients	Logistic ML algorithm	[172]
**Risk assessment of Diabetes**		
25,186 patients	Regularized and weighted RSF	[177]
273 678 patients	DeepSurv and RSF	[178]
11,000 persons	RF classifier	[179]
15,928 Chinese adults without diabetes at baseline (**DRYAD**)	XGBoost	[180]
1,832, 270 cases of type 2 diabete	Gradient boosting decision tree algorithm and LightGBM	[181]
6025 participants	Naive Bayes approaches and LR	[182]
40,124 patients from the GIANTT database	Ridge logistic regression, logistic regression with backward selection, LASSO LR, elastic net logistic regression, and RF	[183]
36,652 eligible participants from the Henan Rural Cohort Study	Classification and regression tree (CART), RF, GBM, LR, SVM, and ANN	[184]
997 subjects with CT scans and contextual EMR scores	Deep neural network	[185]
17,658 in-patients with diabetes who underwent 32,758 admissions	LR and XGBoost	[186]
10,464 diabetic patients	LR	[187]
34 patients	Aggregation method	[188]
1647 obese, hypertensive patients	KNN and RF	[189]
800 T2DM patients	BN, ANN, CRT, CHAID, discriminate, QUEST, and ensemble models	[190]
112 patients over a range of 90 days	LR and RF	[191]
**Dietary and insulin dose modifications**		
23 adults with newly diagnosed T2DM	Algorithm-based personalized postprandial-targeting	[194]
100 adults under different realistic scenarios lasting three simulated months	Reinforcement learning	[195]
**Diabetes management**		
12 subjects with T1DM	Linear discriminant analysis, ensemble learning, Gaussian process regression, KNN, SVM, decision trees, and deep neural networks with LSTM	[199]
16,848 inpatients receiving subcutaneous insulin who achieved target blood glucose control of 100–180 mg/dL on a calendar day	A combination of RF, regularized regression, and GBT	[200]
110 pediatric patients with T1DM	RF and quantile regression forest	[202]
68,274 samples collected from 1119 subjects	Deep learning	[204]
116 subjects	SVM	[205]
**D1NAMO dataset** contains data for nine patients with T1DM	RNN-LSTM	[207]
70 participants with T1DM	K-means clustering	[208]
100 subjects over a two-month scenario	XBM	[209]
250 24 h CGM plots	SVR and multilayer perceptrons	[210]
15 patients with T1DM	SVR	[211]
3 real subjects	Multiple boundaries and domain-based, density-based, reconstruction-based, and unsupervised models	[212]

## Common machine learning and deep learning algorithms

### Logistic Regression

Logistic Regression (LR) is commonly used to allocate observations to a distinct set of classes. It transforms its output by employing the logistic sigmoid function to evoke a probability amount. Binary (e.g. sickly or healthy) and multi-linear functions failsClass (e.g. healthy, pre-disease, or sickly) are two common types of LR [19]. The LR can be called a linear regression model, however, it employs a more complicated cost function as the ‘sigmoid or logistic function’. In ML, the sigmoid is utilized to map predictions to probabilities. The hypothesis of LR is relied on the limitation of the cost function between zero and one, while the linear function may have an amount less than zero and higher than one. Moreover, LR can overestimate the prediction accuracy owing to sampling bias. It also may lead to unfavorable accuracy in presence of intricate relationships between input variables [33].

### Decision Tree

Decision Tree (DT) algorithm is a non-parametric algorithm that can be employed for both aims of regression and classification problems. The tree can be defined by two concepts, namely decision nodes and leaves [34]. The leaves are the decisions or the eventual results. The decision nodes are the places where the data is split. The root of the tree is considered the starting point for forecasting a class label for a record. Then, the amounts of the root attribute are compared with the record’s attribute, and the branch corresponding to that amount will be followed and jump to the next node. One of the limitations of DTs is that they are widely labile in comparison with other decision predictors. A small alteration in the data may lead to a great alteration to the structure of the decision tree and a different outcome.

### Gradient Boosting Machine

Gradient Boosting Machine (GBM) is an ensemble decision tree based on either regression or classification tree models. A GBM can combine the predictions from various DTs to give the ultimate prediction model. GBM is one of the strong approaches for constructing predictive models. Boosting assists in improving the tree’s accuracy. The gradient boosting approach generalizes tree boosting to increase speed and also interpretability. In this algorithm, the models are sequentially constructed. Moreover, it is tried to decrease the faults of the prior model by creating a new model on the residuals or the errors of the former model. Gradient Boosting Regressor (GBR) is used for the continuous targets while Gradient Boosting Classifier (GBC) is implemented for the classification problems. The purpose is to reduce the loss function by appending the feeble learners utilizing gradient descent. The only discrepancy between the above-mentioned algorithms is the loss function. GBM is highly flexible and customizable to any special data-driven role. Moreover, the GBMs are successful in different data-mining and machine-learning problems [35]. However, generally, boosting algorithms can lead to overfitting the outliers. Gradient boosting algorithm is also time-consuming and computationally costly.

### Extremely Gradient Boosting (XGBoost)

Extremely Gradient Boosting (XGBoost) is an optimized GBM learning library that employs DT as the base appraiser. The trees are constructed utilizing residuals, not the real class labels. The base appraisers are regarded as regression trees instead of classification trees because the residuals are continuous. The maximum size of the trees can be determined to downgrade the risk of overfitting. The learning rate is applied to scale the quantity of each tree. The probability in each step is the one of an event computed at a prior stage. The probability of 0.5 is considered the primary probability, which is employed to construct the initial tree. For the further trees, the former probability is measured based on primary prediction and predictions from all previous trees. The similarity score is calculated for each leaf and then the Gain is computed. The node with the highest Gain is then selected as the best cleavage for the tree [36]. The disadvantages of XGBoost are its weak performance on unstructured and sparse data and its sensitivity to outliers because each classifier should resolve the errors in the predecessor learners.

### Adaptive Boosting

Adaptive Boosting (AdaBoost) is an ensemble method. A decision tree with only one split is the most popular algorithm employed with AdaBoost. These trees are sometimes called by another name, decision stumps. This algorithm begins by constructing a decision stump and then ascribing identical weights to the whole of the data points. Afterward, it enhances the weights for the misclassified points and lowers the weights for the corrected classified points. The points with higher weights are given more significance in the further model. It can lead to improving the predictions that are constructed by the initial stump. Until achieving a lower fault, training models will be held. In AdaBoost, alteration of the base estimator is possible for developing the requirements [37]. When AdaBoost is considered a generalized additive model, the logistic regression can be applied for the cost function which is called the LogitBoost algorithm. AdaBoost needs high-quality data and is also very susceptible to noise and outliers in data. The speed of AdaBoost is also lower than the XGBoost algorithm.

### Support Vector Machine

Support Vector Machine (SVM) is a powerful algorithm in which a hyperplane with an N-dimensional space exists corresponding to the number of features for classifying the data points. Hyperplanes denote the decision boundaries used to classify the data points. The dimension of each hyperplane is defined regarding the number of variables in the dataset. Support vectors are data points that are near the hyperplane and impress the location and orientation of the hyperplane. In general, the output of the linear function is taken in SVM. The output > 1 is placed in one class and the output of -1 is categorized in another class. This means that the reinforcement range of amounts (1, -1) acts as a margin. In the SVM approach, the margin of the classifier and the hyperplane should be maximized. The loss function that assists in the maximization of the margin is hinge loss. A regularization parameter is also used to balance the loss and margin maximization. Then, the partial derivatives concerning the weights are calculated to identify the gradients and also to update the weights. In the absence of misclassification, the model predicts the corrected class of data, and the gradient is updated by the regularization parameter. In the presence of misclassification, the model predicts wrongly the class of data, so the loss accompanied by the regularization parameter should be considered to accomplish gradient update [38]. SVM employs kernel functions to accomplish classification on non-linear data [39]. The utilization of SVM in regression is known as Support Vector Regression (SVR). The limitations of SVM are computationally costly for complex and large datasets, low performance in noisy data and when the number of variables is more than the number of training data samples, and classifying only two classes by the generic algorithms [33].

### Random Forest

Random Forest (RF) is an ensemble of DTs that are generally trained with the “bagging” procedure. Each tree in the random forest represents a class of forecasting. Among them, the one with the utmost vote is considered the model’s prediction. The hyperparameters in RF are employed to make a rapid model and enhance the predictive power of the model. RF could be employed for both regression and classification problems. The major restriction of RF is due to the large quantities of trees that slow the algorithm and it can be ineffectual for real-time predictions [40]. It is also computationally expensive and is sensitive to trivial alterations in the data.

### Least Absolute Shrinkage and Selection Operator Regression

Least Absolute Shrinkage and Selection Operator (LASSO) is a regression approach that carries out both regularization and feature selection to boost the accuracy of prediction and interpretability of the resulting model. It is a common kind of regularized linear regression comprising a penalty. It results in a decrease in the coefficients for those input features that do not assist the prediction task. This penalty also causes the assigning of zero values for some coefficient values and excluding some input features from the model. In addition, this algorithm reduces the absolute amount of the regression coefficients and ameliorates the accuracy of the designed linear regression models [41]. The main drawback of the LASSO method is that the regression coefficients can be unreliably interpretable in terms of independent risk factors. This is due to that it relies on the best-combined forecasting, not on the accuracy of the approximations.

### Bayesian Networks

Bayesian Networks (BNs) are graphical models comprising information about the probability of relevance between features and assisting decision-making. The probability relationships can be proposed by the users or can be updated employing the Bayes theorem. The dependency of the inter-variable is shown by nodes and directed arcs (conditional relationships) in the shape of a Directed Acyclic Graph (DAG). Generally, two factors are implicated in learning a BN including structure learning, which contains detecting the DAG, and also parameter learning which includes learning regards to conditional probability distributions. The DAG search and K2 algorithms are the two most common approaches for specifying the structure of the DAG. These algorithms ascribe equivalent previous probabilities to the DAG structures and seek the structure to enhance the probability of the data. This probability is called the Bayesian score. It is important to combine previous information about causal structures in the process of parameter learning [42]. The automatic learning of the graph structure of a BN is a problem that is followed by machine learning. One of the main limitations of BN is that there is no generally accepted approach for building a network from data. It leads to the exploitation of only casual impressions by BN that are detected by its programmer.

### K-Nearest Neighbor

The K-Nearest Neighbor (K-NN) algorithm is an easy-to-accomplishment machine learning algorithm that can be employed to resolve both regression and classification problems. K-NN attempts to forecast the true class for test data by computing the distance between them and all the training points. Next, the K number of points that are nearest to the test data would be chosen. The K-NN algorithm computes the probability of the test data appertained to the classes of ‘K’ training data and selects the class with the highest probability. In the case of regression, the amount is the average of the ‘K’ selected training points [43]. One of the limitations of K-NN is that it doesn’t make good forecasting for infrequent classes such as new diseases. It is also costly in terms of memory and time. In addition, Euclidean distance is very susceptible to magnitudes, so features with high magnitudes will weigh more than others with low magnitudes.

### Artificial Neural Networks

Artificial Neural Networks (ANNs) are a series of algorithms that are developed to identify patterns and classifications. ANN is identified as a feed-forward neural network since inputs are considered just in the forward orientation. ANN comprises the input, hidden, and output layers. The input layer embraces the inputs, the hidden one surveys the inputs, and also the output one generates the outcome. While each layer endeavors to learn the specified weights, ANN can learn every nonlinear function. ANNs are commonly recognized as general function estimators. One of the major causes of generic estimation is the activation function, which presents nonlinear attributes to the network. It aids the network to learn any complex connection between output and input. ANNs are implemented for classification and clustering. Particularly, they are used to categorize the unlabeled data based on resemblances among the inputs. In addition, they can help for classifying the labeled data [44, 45]. Some of the limitations and disadvantages of ANNs are as follows: high computationally cost to construct and train a complex network, non-approachability of the decision-making process for the user, non-generalizability from limited training data, requiring the pre-processing of the independent variables or predictors, and proneness to overfitting [33, 46].

### Recurrent Neural Networks

Recurrent Neural Network (RNN) models are a popular kind of deep learning neural network. RNN passes the backpropagation process through time for learning. It can be developed for handling the data when it loops back the previous information. The output originating from the past state provides the input to the current one. It means that the current state is updated in each time step to obtain the renewed information for arranging the time-dependent sequence data. The impressive success of RNN comes back to generating the sequence data that are supplied from many applications in dynamic systems such as time series, natural language processing, and speech recognition [47]. The limitations of RNN methods are as follows: the speed of calculations is low, training of RNN models may be tough, the model cannot process very long sequences if using relu and tanh as activation functions, and there are gradient disappearing and exploding problems.

### Long Short-Term Memory Networks

Long Short-Term Memory (LSTM) networks are a category of RNNs that is able to learn order dependence. They comprise four neural networks and multiple memory blocks called cells in a chain structure. An ordinary LSTM unit includes a cell, an input gate, an output gate, and a forget gate. Three gates control the amount of information in and out of the cell since it remembers amounts over arbitrary time intervals. The LSTMs can hold information for a long time by default. The LSTM algorithm is well adapted to classify, analyze, and forecast time series of indistinct duration. However, LSTMs need a long time, resources, and high memory to train. The easy overfit is another limitation of LSTMs.

### Gated Recurrent Unit

Gated Recurrent Unit (GRU) is a progressed format of the standard RNN. GRUs are the same as LSTM since use gates to control the flow of information. GRU has a simpler architecture than LSTM [48]. The update gate determines the value of prior information that needs to pass along the further state. The Reset Gate is responsible for the short-term memory of the network. It is employed from the model to determine how much of the prior information is needed to neglect. First, the reset gate stores related information from the past time stage into new memory content. Next, it multiplies the input vector and hidden state with their weights. Then, it computes element-wise multiplication between the previously hidden state and the reset gate. After summing up the above stages, the non-linear activation function is used and a further sequence is produced [49]. GRU models confront some problems including low learning efficiency and slow convergence rate, leading to too long training time and even under-fitting.

### Convolutional Neural Network

Convolutional Neural Networks (CNNs) are one of the most popular DL models that work with backpropagation neural networks. The main success of CNNs is in the field of image processing and image recognition which are adapted to analyze visual imagery specifically with image or video data. The architecture of CNN is comprised of a convolutional, pooling, and fully connected layers. In the convolutional layer, the feature extraction occurs by the kernels. The pooling layer carries out a dimensionality diminution to minimize the network computational effort. The fully connected layer plays the classifier role with non-linear classification. By repeating the convolution-pooling sequences multiple times, the system is deepened and the desired accuracy can be obtained. The traditional NNs generally utilize the full connections between the layers leading to over-fit of train data and intensive time computations. Unlike NNs, CNNs avoid overfitting and general multiplications by using particular layers and consequently provide faster computations [50, 51]. However, CNN does not encode the orientation and position of objects. It is unable to be spatially invariant to the input data. Finally, it needs a large amount of training data.

### Deep Reinforcement Learning

Deep reinforcement learning integrates artificial neural networks (deep learning) with a framework of reinforcement learning (RL) to learn software agents how to achieve their objectives. Reinforcement learning algorithms can start from a blank slate, and under the correct directions, reach superhuman performance. Different penalizes and rewards are considered for these algorithms when they make mistakes and the right decisions, respectively– this means reinforcement. The common steps of RL are as follows: (i) observation of the environment; (ii) selecting a strategy; (iii) acting based on the chosen strategy; (iv) receiving a reward or penalty; (v) learning from the experiences and improving the strategy; (vi) iterating to reach an optimal strategy. Three approaches for reinforcement learning are Policy-based. Value-based, and Model-based learning. Model-based RL utilizes experience to build an internal model of the transitions and instant results in the environment. Proper actions are then selected by exploring or planning in this world model. On the other hand, model-free RL uses experience to learn straightly one or both of two simpler quantities which can reach the same optimal behavior but without approximation or use of a world model. One of the most used learning models is Q learning which is a values-based and off-policy learner algorithm. Value-based algorithms update the value function according to an equation, especially the Bellman equation. Off-policy algorithms learn the value of the optimal policy apart from the agent’s actions. The ‘Q’ in Q-learning refers to quality. Quality indicates how beneficial a given function is in obtaining some future reward. There are some challenging drawbacks and limitations with RL: (i) too much reinforcement can lead to an overload and attenuate the outcomes; (ii) it is not a preferred model for simple problems; (iii) it needs a large number of data and contains plenty of computation; (iv) its preservation cost is high.

## The application of ML and DL models for the management, prediction, and detection of diabetes

### Early diagnosis and prediction of diabetes

Early diagnosis and forecasting of diabetes occurrence through the measurement of several baseline factors could diminish the complications in the future. However, the slight incidence rate of diabetes causes a major challenge in the accurate prediction of diabetes class against non-diabetic one. To overcome this difficulty, various ML and DL algorithms have been developed which are overviewed in the following sections for each diabetes type.

#### T2DM

One of the interesting methods for diabetes detection in Traditional Chinese Medicine (TCM) is characterized based on tongue features. It is performed by analyzing the extracted parameters from the panoramic tongue images such as tooth markings, shape, texture, color, and fur [52]. An experienced person as a specialist is required to do these visualized diagnosis processes. The yellow tongue is defined as a sign of diabetes [53]. For predicting the T2DM risk, an ML model was arranged in a combination with TCM. To this aim, TDA-1 Tongue Diagnosis Instrument was extended by a research group at Shanghai University of TCM to gather the tongue images. This information in association with the common knowledge and laboratory outcomes of the physical checkup was prepared to propose a model. Through the developed model, the texture and color characteristics of the tongue were computed by the Tongue Diagnosis Analysis System (TDAS) which was also improved by the same research group. TDAS performed the tongue diagnosis and the tongue division through the human-computer interplay. Afterward, it could automatically measure the texture and color features. In addition, deep learning techniques were also employed. To train DL models, Mask Region-Based Convolutional Neural Network (Mask R-CNN) was implemented because of its characteristics for the fast object detection of an image with the high-quality. Specifically, it carried out the instant semantic segmentation that led to being worked as a diagnostic support tool. Through image detection, Mask R-CNN could eliminate the background of the raw tongue images and had superior pre-trained models, swift training speed, and remarkable accuracy. Then, eight classical ML models, including LR, RF, Naive Bayes (NB), SVM, XGBoost, ANN, K-nearest neighbor (K-NN), and DT were applied to fully detection of the inherent regularities. To find the best classification impact, the model fusion was carried out by the stacking technique. The stacking process comprised two steps. In the first part, the operation of eight independent models was evaluated by using ten-fold cross-validation. The prediction outcomes constructed the further training data. Simultaneously, the test dataset was predicted and the next data test was built. In the subsequent step, a second test dataset was employed to fit the training data matrix that was integrated into step one using the LR algorithm. Finally, it could test the data matrix for the ultimate outcomes. To create a convenient and noninvasive diabetes risk forecasting model, tongue features and generic details were accomplished to assess the non-invasive stacking model. Moreover, DL methods including XceptionResNet50, DenseNet121, Vgg16, Vgg19, and InceptionV3 were used to train the model. The stochastic gradient descent was performed for optimization, while the loss function was cross-entropy. The best accuracy was obtained for the non-invasive stacking model and ResNet50 at 71% and 69%, respectively. In the critical BG group, the AUC was acquired at 0.84 for the stacking model and 0.88 for ResNet50. In the high BG group, the AUC was achieved as 0.87 and 0.80 for the non-invasive stacking model and ResNet50, respectively.

In another study [52], a deep autoencoder learning algorithm with CNN was developed to exploit deep variables from panoramic tongue images. Then, these parameters were trained with a deep Radial Basis Function Neural Network (RBFNN) classifier algorithm. The suggested model showed a better classification performance rather than the other models. The training accuracy of the ResNet50-RBFNN model was obtained at 92.3%.

The possibility of predicting diabetes could be found from the special patterns of body fat distribution using Magnetic Resonance Imaging (MRI) [54]. For constructing a powerful model, a novel ML method on more than 2000 whole-body MRI image data sets was developed. For this research, a network was constructed corresponding to the DenseNet architecture. The 3-dimensional volumes were used as the input. The first layer included a completely connected convolutional layer with a kernel size of 5 and 8 convolutional filters. The pooling layer was then applied to reduce the dimensions of the mediator feature maps in order to improve the computational performance. The output was entered into the initial dense block. Transition layers and dense blocks were consecutively appended to process the input. After the eventual transition layer, the activation maps were widened to a 1D array and transferred to 3 consecutive densely connected layers with dropout. The dimension of the resulting dense layers was 1 × 128 units, and it was considered the embedding layer for embodying the low-dimensional delegations of MRI voxels as “learned” by the neural network. The embedding layer was employed to forecast the favorable target labels and the unsupervised clustering analysis. Further dense layers were appended to the embedding layer to predict output nodes. Models were trained for age, sex, HbA1c, insulin sensitivity, BMI, prediabetes, and the occurrence of diabetes. The AUC was found at 87% for T2DM discernment and 68% for prediabetes, which were higher than the conventional models. Moreover, the lower visceral abdominal was found a critical region in the classification of diabetes.

To determine the undiagnosed T2DM in adults, the nutritional markers were found by ANN, LR, and RF models [55]. To overcome the impact of the class imbalance, resampling algorithms containing Random Oversampling Examples (ROSE), minority class over-sampling, and Synthetic Minority Oversampling Technique (SMOTE) were applied. In this process, four models were constructed with (1) original data, (2) oversampling, (3) SMOTE, and (4) ROSE per each algorithm. Undiagnosed T2DM was identified by a negative reply to the query “Have you ever been told by a doctor that you have diabetes?” and a positive glycaemic reply to one or more of the 3 detection tests FPG > 125 mg/dl, HbA1c > 6.4% or 2-hr post- oral glucose tolerance test (OGTT) > 200 mg/ dl. As a result, the prevalence of undiagnosed T2DM was determined as 5.26%. The best models classified 39 markers including 11 unique markers by the logistic technique and 28 via one or more of the three best-performing ensemble/non-linear models. They comprised 4 diet-associated, 9 socio-behavioral, 2 anthropometry-based, and 14 nutrient-based markers. The best-performing approach was a LR method on original, unbalanced training data without any resampling with an AUC of 0.74, a sensitivity of 0.77, a specificity of 0.61, and an accuracy of 0.62. This study suggested personalized clinical nutrition like the risk-stratified nutritional recommendations and the early preventive plans that were aimed at high-risk people as well as the nutritional handling of individuals with T2DM.

The results of a systematic review revealed that the optimal performance for the prediction of T2DM is obtained using tree-type models [56]. However, they need complementary approaches to decrease the dimensionality and balance data by choosing the optimal features. Therefore, SVM and KNN have been commonly preferred for prediction. Moreover, it was concluded that a minimum of three parameters from the confusion matrix (accuracy, precision, specificity, sensitivity, and F1-score) and the AUC should be calculated to reduce the heterogeneity in the validation parameters.

To predict T2DM incidence in obese women, ANN-based on anthropometric and adipocytokines variables were applied [57]. The Separability-Correlation Measure (SCM) was first utilized to select the substantial features. The FBS and HbA1c were found as the best discriminators; homeostatic models assessment as the moderate discriminator; adiponectin, visfatin, and insulin as the weak discriminators of diabetic women. The more chosen variables were apelin, visfatin, HbA1c, FBS, adiponectin, and total cholesterol. Moreover, the subsets of these parameters containing apelin, FBS, HbA1c, and visfatin were found to be the remarkable features that could perform the best discrimination between diabetic and non-diabetic groups.

Electronic Health Record (EHR) is a digital copy of a patient’s paper chart that makes information accessible securely and instantly for users. A novel prognostic method for the prediction of T2DM based on EHR was developed without employing the current invasive approaches [58]. The methodology was based on RF frameworks with data enrichment utilizing the temporal variables. The AUC of 84.2% was obtained with an RF classifier, 83.1% when temporal features were used, and 83.7% after using both the feature selection and the temporal features. It was concluded that pathology prediction was feasible and effective employing the information of a patient’s progression over the years and without utilizing invasive approaches.

The OGTT was also employed to create a predictive model for T2DM utilizing SVM [59]. The insulin concentrations were also considered before glucose intake at 30, 60, and 120 min, plasma glucose, personal information like ethnicity and age, as well as the BMI. All accessible combinations of the 10 best-ranked variables were employed to produce SVM-based models. The outcomes revealed that the glucose levels in plasma and the information obtained therefrom could provide the uppermost predictive performance for the future progress of T2DM. Moreover, demographic features and insulin ones did not present the additional performance advancement for diabetes forecasting. The average accuracy and sensitivity were acquired as 96.80% and 80.09%, respectively.

A novel adherence diagnosis method that employed deep learning approaches was developed for T2DM cases, based on simulated Continuous Glucose Monitoring (CGM) signals [60]. Various classification models including LR, CNN, Multi-Layer Perceptrons (MLPs), and ensembling methods were evaluated. The foremost performing models were acquired for CNN with accuracy of 77.5 ± 1.4% and MLP with the accuracy of 72.5 ± 3.5%.

The Artificial Immune Recognition System (AIRS) was another approach that was used for medical classification problems [61]. AIRS utilizes resources contest, clone choice, maturation, mutation, and generation of memory cells for the advancement of forecasting modeling. In addition, AIRS2 is a more effective version of the AIRS method. The modified AIRS2 called MAIRS2 was considered a developed K-NN algorithm. Using the AIRS2 learning algorithm, the size of the dataset named Memory Cells Pool was decreased. Then, the fuzzy K-NN was applied to dominate the constraints of the K-NN classifier by ascribing a class membership to each case. The highest classification accuracy was acquired as 89.10% and 82.69% for MAIRS2 and AIRS2, respectively.

Diabetic Sensorimotor Polyneuropathy (DSPN) is a remarkable consequence of diabetes mellitus, so early diagnosis or prediction of DSPN is essential for preventing foot ulcers and neuropathic pain [62]. Three machine learning methods including SVM, XGBoost, RF, and their combinations were considered to predict four classes containing normal, possible, probable, and confirmed based on the electrophysiological and clinical characteristics of the doubtful DSPN. RF showed the best AUC (0.82); and the average values of the International Federation of Clinical Chemistry (IFCC), serum glucose, albumin levels, and HbA1c were found as the major predictors. Therefore, it was concluded that ML techniques could help in predicting the DSPN and electrophysiological analysis in T2DM.

Three potent machine learning algorithms including XGBoost, DNN, and RF were applied to forecast the forthcoming occurrence of T2DM based on the biochemical, demographic, and anthropometric measures [63]. Furthermore, three strategies containing cost-sensitive learning, changing threshold, and sampling were used to overcome the imbalance challenge in the diabetes classes. Weighing and changing thresholds caused a reduction in the training time, enhancement of performance, and increased prediction accuracy in the minority diabetes classes. Although sampling led to better performance, it was not found as the best solution to resolve the imbalance distribution between healthy and diabetic classes.

XGBoost, SVM, LR, RF, and ensemble algorithms were also utilized to construct models to predict T2DM incidents in the following year (Y + 1) by employing the variable values in the ruling year (Y) [64]. Before constructing the prediction model, the major features were initially chosen utilizing a data-driven feature selection approach. It contained an analysis of a chi-squared test, variance (ANOVA) test, and the recursive feature elimination methods. The elements including Triglycerides (TG), HbA1c, FPG, gamma-GTP, BMI, uric acid, age, smoking, sex, Physical Activity (PA), drinking, and family history were selected as the variables. The prediction models accurately anticipated the normal (non-diabetic), prediabetes, or diabetes in the Korean population. The accuracy was identified as between 71% and 73%. Moreover, the ensemble models had better proficiency than the single models. The performance of the prediction models was improved by incorporating more medical history from the dataset. In another work, the DT and LR models were also used to predict T2DM [65]. As a result, five main predictors including pregnancy, glucose, age, BMI, and diabetes pedigree function were found as the best classifiers. The prediction accuracy was obtained as 78.26%.

T2DM in youth is more challenging to therapy because of a more quick reduction in the beta-cell function, and also the appearance of onset of complications. The NB, LR, LogitBoost, and DT methods were tested based on HbA1c, FPG, and 2hrPG to improve preDM/T2DM screening performance [66]. The results showed that they were statistically equivalent to or better than the screening guideline. The F-measure and specificity were obtained as 0.005 and 0.225, respectively.

To early predict T2DM by employing lifestyle indicators, various ML algorithms using ensemble methods including Boosting, Bagging, and Voting were employed [67]. Boosting converts weak learners into strong learners, in which weak classifiers are combined to constitute a strong model to ameliorate the predictive abilities of the eventual model. The base learning classifier is used multiple times to produce a novel forecasting rule. The steps will be performed n times iteratively and then the boosting method will incorporate the outcomes from weak learners and transform them into a single strong prediction model. The bagging method follows the bootstrap aggregation approach and creates several training sets for model-building purposes. After the construction of disparate training sets, different models are applied to the resampling process with an ensemble structure. Eventually, the outcomes of learners are aggregated to make the ultimate divination. In the voting method, the forecasting of base learners is aggregated to construct new meta-variables for final prediction.The output of base classifiers is combined based on the most votes and weighted approaches. Among all the models, the Bagged Decision Tree (BDT) algorithm achieved the highest testing accuracy rate of 99.14% followed by Stochastic Gradient Boosting (98.45%,), RF (93.63%), Extra Tree (91.41%), Adaboost (89.69%), and Voting classifiers (89.51%). However, according to the number of misclassifications on the test dataset, BDT obtained the lowest rate of 0.86% and Voting obtained the highest rate of 10.49%.

A patient network-based model containing the existing relationships among health situations for a category of subjects diagnosed with a similar disease using the graph theory was developed to predict T2DM [68]. For this purpose, a bipartite network graph whose vertices can be separated into two independent subsets was utilized to provide the diseases that a patient faces over time. Eight ML models including Naive Bayes, SVM, LR, KNN, RF, XGBoost, DT, and ANN were used to predict the T2DM risk. The RF model led to better results. In addition, closeness and eigenvector centralities as well as patient age were determined as the significant variables for the model.

In order to predict the future incidence of T2DM following pregnancy in women, an XGBoost model based on parity, age, gestational age at delivery, gravidity, glucose challenge test (GCT), oral glucose tolerance test results, OGTT, and birthweight was constructed [69]. The prediction model led to an AUC of 0.85 and an accuracy rate of 91%. The most predictive parameters were neonatal birthweight and the age at the index pregnancy.

A gradient boosting decision tree model was developed to predict the occurrence of T2DM 5 years ahead [70]. The model achieved a test AUC of 80.26 and was robust to immigration status, sex, area-level marginalization with regard to race/ethnicity and material deprivation, and low contact with the health care system.

A method called average-based weighted objective distance (AWOD) was developed for the prediction of T2DM [71]. This approach uses information based on average amounts of the expected amounts and acceptable levels to prioritize factors naming as weighing factors. AWOD has three main stages: i) determining important levels for weight calculation including the expected level and an acceptable level. Afterward, the weights for both significant and negligible factors are computed. Finally, AWOD is specified for the prediction. The AWOD-based approach provided 98.95% accuracy which was more accurate than RF, SVM, K-NN, and DL.

The Q-learning algorithm was used for the early detection of T2DM based on several variables such as glucose level, BMI, and age [72]. In the Q-learning algorithm, a set of q-values are approximated by employing an agent and a set of states. The agent will receive positive/negative rewards for each state pair. The agent maximizes the negative/positive reward in the long-term process by learning optimal policy elections for various unique states. The proposed model produced an off-policy-based RL and made the learning agent to identify an optimal policy for the variables. It achieved a better accuracy rate (84%) than the K-NN and DT algorithms.

#### T1DM

An XGBoost model was developed to recognize T1DM subjects misdiagnosed as T2DM using Ambulatory Electronic Medical Records (AEMR) data [73]. The model identified BMI/weight, age, HbA1c/blood glucose values, and therapy history as top predictors of misdiagnosis. The precision of the model at low levels of recall (10%) was 17%, in comparison with the < 1% occurrence rate of the misdiagnosis at the time of the first T2DM. This algorithm could diminish misdiagnosis of adult-onset T1DM.

#### GDM

The earlier diagnosis of GDM is important for barricading or remarkably decreasing the risk of detrimental pregnancy outcomes [74]. A gradient-boosting machine learning model constructed by decision-tree base-learners was applied based on the electronic health records. The models anticipated GDM with high accuracy even at the pregnancy beginning (AUC = 0.85) more than a baseline risk score (AUC = 0.68). The results were confirmed in both a geographical validation set and a future validation set. Eventually, a model was introduced based on the nine queries that a patient could reply to them for determining the early-stage intervention in high-risk women (AUC = 0.80). In another study, eight ML approaches including RF, logistic, XGB, GDBT, LGB, AdaBoost, and Vote as well as LR with RCS and stepwise logistic regression were examined to predict the incidence of GDM [75]. The maternal demographic specifications, the medical history, and also the laboratory amounts at early pregnancy were selected as the predictors. Variables were trained by discrete ML models and traditional LR models. In the validation dataset, the LR and ML models were carried out moderately (AUC: 0.59–0.74). Overall, the GBDT model had the best performance (AUC: 0.74) among the other ML models. BMI, HbA1c, FBS, and TG were found to have a strong contribution to GDM. A cut-off of 0.7 had a positive predictive amount of 93.2% and a specificity of 99%. Moreover, it was realized that machine learning approaches did not have superiority over LR in forecasting GDM. In another study, Light Gradient Boosting Machine (lightGBM) and SVM were utilized to develop the first 19 weeks risk prediction model for GDM [76]. The predictors included blood routine, coagulation function, and hepatic and renal functions were used. It was found that a cutoff of Activated Partial Thromboplastin Time and Prothrombin Time can accurately forecast GDM with a specificity of 99.47% and sensitivity of 88.3% (AUC = 94.2%). If only renal and hepatic functions were used, a cutoff of FBG and direct bilirubin with a specificity of 90.0% and sensitivity of 82.6% (AUC: 91.0%) were specified. And a negative correlation between prothrombin time and patients with the activated partial thromboplastin time was identified. A negative correlation with direct bilirubin and a positive correlation with FPG could neglect the coagulation function test. As a result, the outcomes could disclose the feasible functions of prothrombin time and activated partial thromboplastin time as biomarkers for the forecasting and early detection of GDM.

GDM is commonly affirmed with an OGTT during 24 to 28 weeks of gestation [77]. A cost-sensitive hybrid model (CSHM) and five machine learning approaches including BN, LR, chi-squared automatic interaction detector (CHAID) tree, SVM, and NN were utilized to construct the predictive models based on EHR. The accuracy of positive samples was (62.16%), however, the outcomes proposed that the wide majority (98.4%) of those forecasted positive cases were real positives.

A novel analytical platform (Reverse Engineering and Forward Simulation [REFS]) was developed to construct a prediction model for the development of prediabetes or T2DM using the EHR information [78]. REFS was based on a Bayesian scoring algorithm to follow a vast model space and produced a dispensation of risk approximates from an ensemble of prediction models. The model predicted the progression to T2DM (AUC = 0.76). Models of development to T2DM included primarily appointed risk factors including lipid disorders, triglycerides, socioeconomic factors, blood pressure, blood glucose, and hypertension. While, models of the development of prediabetes contained novel factors including C-reactive protein, alanine aminotransferase, high-density lipoprotein, and body temperature (AUC = 0.70).

Obesity and body fat dispensation are substantial risk factors for type 2 diabetes [79]. To forecast the FPG situation, a composition of different measures was considered. Based on thirty-seven anthropometric values, the predictions of the FPG using the NB classifier and LR were compared. The AUC was obtained as 0.739 and 0.741 for females as well as 0.686 and 0.687 for males using LR and NB classifiers, respectively. The outcome revealed the superiority of a combination of anthropometric measures over case measures alone in both males and females.

AIRS was also used to predict T2DM following GDM [80]. Despite the dataset having imbalanced data, the classification recall reached 62.8%. To develop a simple model to forecast GDM in early pregnancy, biochemical biomarkers and multivariate Bayesian logistic regression using the Markov Chain Monte Carlo simulation algorithm were applied [81]. At first, the predictive maternal factors were chosen through Bayesian adaptive sampling. From the 8th to 20th week of gestation, FPG levels diminished slightly and TG levels elevated slightly. The risk of GDM was predicted with prepregnancy BMI, maternal age, TG, and FPG with an accuracy of 0.64 and an AUC of 0.766.

In order to construct a preconception-based GDM predictor, game theory concepts were merged with genetic programming (GP)-based automated machine learning (AutoML) [82]. For this purpose, the Shapley additive explanations (SHAP) framework was combined with the GP-based Tree-Based Pipeline Optimization Tool (TPOT) to find significant features and choose optimal supervised machine learning models. GP trains the ML problems based on random mutation, crossover, fitness functions, and production to reach optimal solutions. The Shapley amount is the average expected marginal contribution of one player across all accessible permutations of players in game theory. In ML, game players are the variables, and the collective payout is the model forecasting. The SHAP framework supplies local explanations based on the Shapley amounts to find the global model structure. A stacked ensemble model with a linear SVM classifier and GB classifier was achieved utilizing GP. The resulting AUC was 0.93 based on four features (fasting insulin, glycated hemoglobin A1c (HbA1c), triglycerides/HDL ratio, and mean arterial blood pressure). The multivariate logistic regression model also revealed that each 1 mmol/mol rise in preconception HbA1c was positively associated with the appended risks of GDM and preterm birth. Therefore, the control of preconception HbA1c may help avoid GDM and decrease the occurrence of preterm birth.

#### All diabetes types

Some ML models have been presented for the prediction and diagnosis of diabetes regardless of its type. A filter approach based on the DT algorithm (Iterative Dichotomiser 3) was developed to select important features [83]. Two ensemble learning algorithms, RF and AdaBoost were employed for feature selection. Also, it was compared with wrapper-based feature selection algorithms. The superior performance of the suggested approach was due to the various combinations of the chosen feature set. Diabetes pedigree function, plasma glucose concentrations, and Blood mass index were the most substantial major features for the prediction of diabetes. The suggested method achieved 98.2%, 99.2%, and 99.6% with the cross-validation methods of hold out, k-folds, and LOSO, respectively. Furthermore, the proposed approach would impressively diagnose diabetes and could be utilized in an e-healthcare environment. In another study, an ensemble-based framework called eDiaPredict was suggested to predict diabetes [84]. This algorithm employs an ensemble of various ML algorithms including RF, XGBoost, SVM, NN, and DT. Recursive Feature Elimination (RFE) [85] was used to decrease the feature space in the dataset. The ensemble RF as a bagging method with the XGBoost as a boosting approach generated the best outcome by forecasting the diabetic patient with 95% accuracy. It is due to diminishing the bias recursively and identifying the best solution.

To predict the occurrence of diabetes by employing EHR, LightGBM approach was applied [86]. The predictive ability of this approach was compared with the LR model and led to 0.865–0.925 vs. 0.778–0.876 for various datasets. A novel computing scheme was proposed to categorize several types of diabetes, as they need different treatments [87]. It contained two steps: (1) the major features were found from the glucose concentration curve obtained by the CGM system; (2) a model of diabetes parameter regression called double-Class AdaBoost was constructed. The experiments revealed the coincidence rate of the proposed scheme and the clinical selection at 90.3%. In another work, the Gaussian Process (GP)-based classification approach was used employing three kernels including linear, polynomial, and radial basis [88]. The best performance was obtained for the GP-radial basis kernel with an AUC of 81.97%, a sensitivity of 91.79%, and a specificity of 63.3%.

In a study, RF was employed to detect the occurrence of diabetes in a large set of observational data and figure out the potential predictors of diabetes [89]. The full RF model assessed 93 features including demographic, blood biomarker, anthropometric, echocardiogram data, and medical history. In addition, RF metrics of feature significance were utilized to rank features according to their portion of the diabetes forecast. The performance of the RF full model was analogous (AUC = 0.82) to those of more stingy models. The top-ranked features according to RF included FPG, TG, hemoglobin A1C, adiponectin, waist circumference, c-reactive protein, left ventricular mass, aldosterone, leptin, and high-density lipoprotein cholesterol.

A fuzzy c-means-neuro-fuzzy rule-based classifier was developed for the classification of diabetes [90]. The accuracy of the classifier was calculated by the number of correctly identified diabetes records while its complications were computed by the number of extracted fuzzy rules. Experimental outcomes revealed that the suggested fuzzy classifier could reach a good tradeoff between accuracy and interpretability. The contribution of the Fuzzy C-Means (FCM) algorithm with the Adaptive Network-Based Fuzzy Inference System (ANFIS) decreased the learning time and size of models. ANFIS is a particular method in neuro-fuzzy modeling that used neural networks to adapt rule-based fuzzy systems. The best accuracy of the proposed method was acquired as 81.54%.

A non-invasive diabetes diagnosis system was also suggested dependent on the wristband basic Physiological Parameters (PhyP) and Photoplethysmography (PPG) signal [91]. The PhyP and Mel-Frequency Cepstral Coefficients (MFCC) were extracted from 5 s PPG signal parts and then applied as the input for the RF, SVM, K-NN, and XGBoost. This data was used to classify the patients into prediabetes, diabetes, and normal classes. Moreover, a Hybrid Feature Selection (Hybrid FS) approach was suggested to decrease the size of the entrance data. The Hybrid FS-based XGBoost system resulted in a significant accuracy (99.93%) for non-invasive diabetes diagnosis with numerable variables and less computational endeavor. The analysis showed that the PPG signal was a suitable substitute for ordinary non-invasive BG evaluation.

Laboratory information such as sex, age, body mass index, TG, FBS, high-density lipoprotein, low-density lipoprotein, and blood pressure was used to predict diabetes mellitus using GBM and LR techniques [92]. Moreover, the adjusted threshold and the class weight approaches were employed for ameliorating sensitivity. The AUC and sensitivity were 84.7% and 71.6% for the suggested GBM model as well as 84.0% and 73.4% for the LR method, respectively. The performance of the GBM and LR models was better than the DT and RF models. These approaches could be implemented in an online computer program to assist physicians in forecasting cases of the future incidence of diabetes and supplying essential preventive interventions.

The correct diagnosis of the diabetes type is sometimes challenging. To recognition of the prediabetic, T1DM, and T2DM types, RF, SVM, DT, KNN, Bagging, and Stacking algorithms were used. The outcomes showed that integration of Bagging K-NN, Bagging DT, and K-NN, with a K-NN meta-classifier, led to an accuracy of 94.48%. Moreover, 5 variables including sex, nutrition, insulin, antiDiab, and education were found that remarkably impressed the model accuracy [93]. In another work, a Decision Support System (DSS) was developed for the prediction of diabetes based on SVM and RF and fully CNN. The obtained accuracies using RF, SVM, and DL were 83.67%, 65.38%, and 76.81%, respectively. The experimental outcomes revealed that RF was more efficient for diabetes forecasting in comparison with other methods [94].

A Reinforcement Learning-based Evolutionary Fuzzy Rule-Based System (RLEFRBS) was developed for diabetes diagnosis. The proposed model comprised a Rule Base (RB) constructed by numerical data without initial rules and rule optimization. Following learning the rules, the surplus rules were excluded. Afterward, the superfluous conditions in the prior parts were pruned to yield modest rules with higher interpretability. Eventually, a proper subset of the rules was chosen to employ a Genetic Algorithm (GA), and the RB was built. To improve the performance of RLEFRBS, evolutionary tuning of the membership functions and weight adjusting using RL were employed. The accuracy of the suggested model revealed that it could be a proper alternative for the diagnosis of diabetes [95].

A pipeline based on deep learning methods was proposed to forecast diabetic people. It comprised data augmentation utilizing a variational autoencoder (VAE), feature augmentation utilizing a sparse autoencoder (SAE), and a CNN for classification. The input features were glucose or insulin level, the number of pregnancies, blood pressure, and age. After training the CNN classifier in association with the SAE for featuring augmentation, an accuracy of 92.31% was obtained. It was concluded that this pipeline was proper in diabetes diagnosis [96].

A model using hidden layers of a deep neural network model (DLPD) was proposed to forecast the incidence of diabetes in the future and also to specify the type of disease. The dropout regularization was applied to avoid overfitting and the binary cross-entropy loss function was used to reach high accuracy. The best training accuracy of the diabetes-type data set was determined as 94.02% [97].

### Prediction of blood glucose

The level of blood glucose (BG) should be maintained within the normal range (70–120 mg/dL or 3.6–6.9 mmol/L) [98]. Keeping the BG amounts at the desired level in diabetic subjects is challenging because the precise glycemic control employing bolus insulin injections may lead to an elevated risk of having hypoglycemic incidents [99]. Therefore, closed-loop systems and computational methods have been developed to help in controlling BG levels. On the other hand, modeling and controlling are two major challenges to completing diabetes management. Modeling refers to learning a precise BG forecasting model based on several variables and controlling refers to applying a developed model to forecast the BG amount and propose some recommendations. The utilized algorithms for the prediction of BG are critical in the progress of closed-loop insulin delivery and decision support systems for the control of blood glucose in diabetes. Multitask learning is a simplified method for leveraging data from numerous cases while it could still do learning accurately the personalized models [100]. This approach showed stable performance in predictive metrics at both long-term and short-term prediction horizons. The predictive accuracy RMSE of 18.8, 25.3, 31.8, 41.2, and 47.2 mg/dL were obtained at 30, 45, 60, 90, and 120 min prediction horizons, respectively, with around 93% clinically admissible predictions employing the Clarke Error Grid Analysis (EGA). The outcomes showed the success of multitask learning compared with sequential transfer learning. In another study, a multisource adversarial transfer learning method was applied to provide the learning of a feature exhibition that was analogous to the sources [101]. For this purpose, a CNN model was employed. The evaluation was performed by tracking multiple transfer methods. This approach was applicable when data originated from the various datasets, or when there was too small data in the intra-dataset condition. Although the adversarial transfer did not differentiate the patients and datasets, it led to learning a general feature representation contrary to a standard transfer. Only insulin, glucose, and carbohydrates data were used for the forecasting of the future glucose amount. The RMSE was obtained between 18.94 and 19.27 for various datasets.

The Kernel Extreme Learning Machine (KELM) neural network, the Variational Mode Decomposition (VMD) method, and the AdaBoost algorithm were integrated to build a multi-scale blood glucose prediction model so-called VMD-KELM-AdaBoost [102]. The VMD approach was first applied to decompose a series of BG concentrations into a series of intrinsic modal functions (IMFs) with various scales. Afterward, in order to increase the prediction capability of the model, the AdaBoost and KELM neural networks were combined to model and also forecast the decomposed IMFs by VMD. The experimental results revealed that this model could perform the BG prediction (the mean values of RMSE was about 10.14); in Clarke error mesh analysis, the ratio of being in A zone was about 95.7% and the sensitivity was 94.8%.

A Monte Carlo (MC) photon simulation-based model was developed for assessing the concentration of blood glucose through PPG on the fingertip [103]. The MC method was selected for the simulation of a photon in the finger model due to its flexibility in calculating the optical interplays with the biological tissues. The intensities of the discovered photons after simulation with the model were employed to assess the BG concentrations using XGBoost. A heterogeneous finger model with the muscle, fat, skin, and bone layers was developed to propagate photons. Bio-optical characteristics such as scattering coefficient, absorption coefficient, refractive index, and anisotropy were determined at wavelengths of 660 and 940 nm to develop the finger model for photon simulations. The model achieved RMSE 16.1.

To predict BG levels utilizing time-series data of patients with T1DM, autoregression with exogenous inputs (ARX) model, ML-based regression models, and DL models including a temporal convolution network (TCN) and a vanilla LSTM Network were examined [104]. The ARX model obtained the lowest average RMSE for both direct and recursive approaches. No remarkable advantage was found from the ML models in comparison with the classic ARX one, except TCN’s performance. TCN was more robust than BG trajectories with false oscillations, for which ARX over-predicted peak BG and under-predicted valley BG amounts.

DL approaches have been also used for an accurate prediction of CGM. A DL algorithm was developed for glucose forecasting that employed a multi-layer Convolutional Recurrent Neural Network (CRNN) architecture [105]. The model was initially trained on data containing carbohydrates, CGM, and insulin data. The CRNN method contained three sections: a multi-layer convolutional neural network to extract the data variables using convolution and pooling, an RNN layer with LSTM cells, and fully-connected layers. The convolutional layer comprised a 1D Gaussian kernel filter to carry out the temporal convolution. The pooling layers were applied to decrease the features. The eventual output was a regression yield by fully connected layers. The model was able to predict glucose levels with superior accuracy for real patient subjects (RMSE = 21.07 mg/dL for 30-min and RMSE = 33.27 for 60-min). The proposed algorithm was implemented on an Android mobile phone, with a performance time of 6 ms on a phone in comparison with an execution time of 780 ms on a laptop. In another work, the CRNN model was used for the precise prediction of glucose levels in T1DM patients for a 30-minute horizon (MAE = 11.22 [mg/dL] and RMSE = 17.45 [mg/dL]), and for the 60- minute horizon (MAE = 23.25 [mg/dL] and RMSE = 33.67 [mg/dL]) [106].

The CGM measurements are sensitive to sensor faults and it would influence the BG prediction. A novel LSTM-based deep RNN model considering the sensor error was proposed for predicting the BG level [107]. A Kalman smoothing method for the modification of the inaccurate CGM readings was used because of the sensor fault. To this end, the various physiological information including bolus insulin, carbohydrates from the meal, Kalman smoothed CGM data, and cumulative step counts in a constant time interval were considered. The goal was to lessen the diversity between the fingerstick blood glucose measurements and the predicted CGM amounts.

Bluetooth Low Energy (BLE)-based sensors could be applied as a device to trace personal vital signs data [108]. A personalized healthcare monitoring system employs real-time data processing, a BLE-based sensor device, and ML-based approaches to improve the self-managing of chronic diabetes situations. BLEs were used for collecting the vital signs data like blood glucose, weight, heart rate, and blood pressure from the sensor nodes to the smartphones. Moreover, real-time data processing was used to handle a large value of continuously produced sensor data. The outcomes revealed that the suggested real-time data processing and commercial versions of the BLE-based sensors were adequately effective to follow the vital signs data of diabetic cases. Furthermore, ML-based classification approaches were examined and demonstrated that a multilayer perceptron could supply early forecasting of diabetes employing the gainer’s sensor data as input with the accuracy of 77.08% in comparison with 76.69%, 73.04%, 76.04%, and 76.56% for NB, RF, LR, and SVM, respectively. The outcomes also disclosed that LSTM can precisely foresee the future BG level.

A novel framework was suggested to incorporate several algorithms for BG prediction in patients with diabetes mellitus [109]. This framework had an adaptive weight which was specified for each approach where one algorithm’s weight was conversely proportional to the sum of the squared prediction errors. The suggested framework was employed to combine SVR, an Extreme Learning Machine (ELM), and an Autoregressive (AR) model. The new adaptive-weighted algorithm reached the best prediction performance of 92.5%.

Several linear black-box approaches (autoregressive, autoregressive moving average, and autoregressive integrated moving average (ARIMA)) and nonlinear ML procedures (SVR, feed-forward neural network (fNN), regression random forest, and LSTM-NN) were examined to forecast the glucose levels and hypoglycemia. The outcomes revealed that the individualized linear models were more efficient than the population ones. The best linear algorithm (individualized autoregressive integrated moving-average) obtained an accuracy analogous to that of the foremost nonlinear algorithm (individualized feed-forward neural network), with RMSE of 22.1 and 21.5 mg/dL, respectively. For the prediction of hypoglycemia, the individualized ARIMA provided precision = 64% and one incorrect alarm/day in comparison with the foremost nonlinear approach (population SVR) with precision = 63% and 0.5 incorrect alarms/day. Moreover, no remarkable benefits were found when nonlinear techniques were used for a 30 min prediction horizon [110].

Predicting glucose amounts based on food and insulin intake is a hard task that should be done daily for cases with diabetes [111]. In a study, the enhanced variants of grammatical evolution, random forest regression, K-NN regression, and tree-based Genetic Programming (tree-based GP) were utilized to build models. They were then examined to predict the glucose concentrations accompany by the approximation of both future insulin injections and carbohydrate intakes. Two new enhanced modeling approaches for glucose forecasting included (i) diverse grammatical progress using an optimized grammar, and (ii) various tree-based GP utilizing a three-compartment model for insulin and carbohydrate dynamics. The experimental outcomes applying the Clarke error grid metric showed that 90% of the predictions were correct (i.e., Clarke error categories A and B). However, it still produced 5 to 10% of drastic errors (category D) and nearly 0.5% of very critical errors (category E). Several ML-based prediction models such as XGBoost, RF, Glmnet, and LightGBM were applied for the prediction of undiagnosed T2DM that were comparable with the regression models [112]. The accuracy in the forecasting of the FPG level was calculated employing 100 bootstrap iterations in various subsets of data. For the six months of accessible data, a simple regression model was carried out with the undermost average RMSE of 0.838, followed by RF (0.842), LightGBM (0.846), Glmnet (0.859), and XGBoost (0.881). The highest level of variable election stability over time was determined with LightGBM models. The outcomes demonstrated no clinically relevant progress when more advanced prediction models were utilized.

Hypoglycemia is defined as a self-monitored blood glucose value < 70 mg/dL [113]. SVM, RF, NB, and K-NN were examined to construct models. The optimal number of self-monitored blood glucose (SMBG) amounts required by the model was almost ten per week. The specificity of the model for the forecasted hypoglycemia incidence in the next 24 h for patients with T2DM was 70% and the sensitivity was 92%. In the model that combined medication information, the forecasting window was for an hour of hypoglycemia, and the specificity ameliorated to 90%. Hypoglycemia occurs asymptomatic in some patients [114]. The precise clinical decision protection tools are required to recognize susceptible patients for iatrogenic hypoglycemia throughout hospitalization. To this end, RF classification, multivariable logistic regression, Stochastic Gradient Boosting (SGB), and NB were utilized. The SGB predicted the potentially serious results of iatrogenic hypoglycemia within 24 h after each BG measurement. In another study, the XGBoost model was utilized to forecast hypoglycemia employing the dataset of multicenter intensive care unit (ICU) electronic health records [115]. The results revealed the power of the model to predict the incidence of hypoglycemia (blood glucose < 72 mg/dL) during the settlement of patients in the ICU.

Predicting the incidence of postprandial hypoglycemia is a challenge because of the glucose fluctuations occurring around mealtimes [116]. To predict this event with a 30-min prediction horizon, four machine learning models including SVM using radial basis or linear functions, RF, LR, and K-NN were applied. The RF model demonstrated the best performance with an AUC of 0.966, specificity of 91.3%, and sensitivity of 89.6%.

Nocturnal Hypoglycemia (NH) (glucose levels < 50 mg/dL) is a challenging concern for individuals with T1DM, because it may not be diagnosed while sleeping [117]. However, predicting NH before sleep could aid to diminish nighttime hypoglycemia. An SVR model was applied to forecast the glucose before bedtime, the overnight minimum glucose, and overnight NH for individuals with T1DM. The least glucose threshold for declaring NH risk was taken by employing a decision-theoretic criterion to increase the expected net gain. The algorithm anticipated 94.1% of NH events (< 3.9 mmol/L) with an AUC curve of 0.86. In addition, the proposed algorithm could decrease NH by 77.0%.

In another study, thirty-two features based on CGM, insulin, meal, and demographics data from the T1DM cases were measured for three sequential days before the night [118]. The linear discriminant analysis was used to find the optimal feature subset, which led to only one feature subset from four features. The assessment resulted in an AUC of 0.79 leading to a specificity and sensitivity of 70% and 75%, respectively. In another work, SVM and MLP were utilized to predict NH based on CGM data and a PA tracker [119]. The predictions carried out by SVM achieved the best population results, with a specificity and sensitivity of 82.15% and 78.75%, respectively.

A model for foreseeing NH with a 6-hour horizon (midnight-6 am) was built using an RF model [120]. This model showed AUC = 0.75 for late-night (prediction at midnight, looking at 3–6 am window) and AUC = 0.90 for an early night (midnight-3 am). While the fluctuation and the lack of late night blood glucose patterns present predictability challenges, this 6-hour horizon model showed an acceptable performance in forecasting NH.

A novel data-driven method to predict the quality of overnight glycaemic control for T1DM patients was introduced by analyzing commonly collected data including insulin boluses, meal intake, and CGM data during the day-time period [121]. Several machine learning approaches for binary classification were assessed. The best AUC for the prediction of glucose at the attendance of NH was obtained with a window of 18 h employing the extended tree classifier (ETC) and SVM classifiers. However, the sensitivity and specificity were moderate (0.5–0.65) for forecasting glucose at night. Although there was no preferred method, SVM and ETC led to better results in forecasting hypoglycemia and normoglycemia. While random forest classifier (RFC) accomplished better outcomes in predicting nocturnal hyperglycemia.

In another study, six techniques were tested for predicting the glucose values from CGM and also modeling the penalty for errors in various glycemic ranges between 10 and 60 min [122]. These models included linear extrapolation, NNs, last observation carried forward, ensemble methods using LSBoost and bagging with error-weights and without error-weights. The advanced machine learning algorithms showed better performance (MARD: 10.26 − 10.79; the 30-min lead time) compared with the simple modeling (MARD: 10.75–12.97; 30-min lead time). The results disclosed that the use of error weights provided better clinical proficiency for these models.

For short-term NH forecasting in hospitalized patients with T1DM, Logistic Linear Regression with Lasso regularization, RF, and Artificial Neuron Networks algorithms were used. Among them, RF led to the best accuracy. The addition of clinical parameters to CGM data somewhat ameliorated the prediction accuracy and resulted in AUC of 0.97 and 0.942 for 15 min and 30 min prediction horizons, respectively. Proteinuria, basal insulin dose, HbA1c, and diabetes duration were the remarkable clinical predictors of NH [123].

It was studied that reverse engineering and forward simulation (REFS) could be applied to constructing ensembles of generalized linear models to determine some significant predictors [124]. These predictors consisted of hypoglycemia, glycated hemoglobin (HbA1c) target attainment, antidiabetic class persistence, T2DM-related inpatient admissions, HbA1c alteration, and T2DM-related medical costs among patients who were treated for T2DM. The results showed that patients with comorbid situations had an elevated risk of hypoglycemia, with previous anemia (OR = 1.29) and hypoglycemia (OR = 25.61). Other identified risk factors contained sulfonylurea use (OR = 1.80) and insulin (OR = 2.84). High blood glucose ([125 mg/dL vs. 100 mg/dL, OR = 0.47; 100–125 mg/dL vs. 100 mg/dL, OR = 0.53), Biguanide use (OR = 0.75), and missing blood glucose test (OR = 0.40) were accompanied by the declined risk of hypoglycemia.

Although insulin therapy decreased the danger of late-diabetic complications by reducing the average blood glucose, the treatment could result in an elevated occurrence of hypoglycemia in T1DM [125]. A novel pattern classification method was developed to identify hypoglycemic occurrence through retrospective analysis of professional CGM data. The proposed algorithm discovered a full prediction of hypoglycemic events with only one false positive.

The possible incidence of hypoglycemia is the main reason that type 1 diabetes patients did not do exercise [126]. To forecast hypoglycemia at the beginning of physical training, DT and RF models were evaluated. The DT model found two major predictive features for hypoglycemia during the workout including glucose at the onset of exercise and heart rate. The accuracy of the model was determined as 79.55% when the heart rate was higher than 121 bpm within 5 min of working and glucose was less than 182 mg/dL at the beginning of the exercise. The RF model reached a greater accuracy of 86.7% by employing extra variables and higher complexity.

Non-invasive hypoglycemia prediction employing the physiological parameters of electrocardiography signal (corrected QT interval and heart rate) was also proposed for T1DM patients [127]. To boost the diagnosis accuracy, an extreme learning machine (ELM)-based neural network was developed to identify the attendance of hypoglycemia. This method reached a faster training performance in comparison with the conventional neural network learning algorithms and dominated the obstacle of over-fitting. The algorithm relied on the empiric risk minimization theory. The learning process requires one iteration and is capable of eschewing multiple iterations. The specificity and sensitivity of the suggested algorithm for diagnosis of hypoglycemic episodes were determined as 60.0% and 78.0%, respectively. In another study, ELM trained feed-forward neural network (ELM-FFNN) was applied to identify the attendance of hypoglycemic events for T1DM patients [128]. The natural incidence of nocturnal hypoglycemic episodes was related to elevated heart rates and corrected QT intervals. By employing the ELM-FFNN, the specificity and sensitivity for the diagnosis of hypoglycemia were 60% and 78%, respectively.

Anticipating insulin-induced postprandial hypoglycemic events is exigent because the primary cautioning of hypoglycemia simplifies the rectification of the insulin bolus before its execution [129]. The postprandial hypoglycemic event could be decreased by diminishing the bolus size but in favor of elevating the mean of blood glucose. The modified SVM was developed for predicting postprandial hypoglycemia employing two risk-based approaches for 240 min after the bolus/meal. The median sensitivity and specificity were found as 71% and 79% for level 1 hypoglycemia (glucose ≤ 70 mg/dL (3.9 mmol/L) and glucose ≥ 54 (3.0 mmol/L)), as well as 77% and 81% for level 2 hypoglycemia (glucose < 54 mg/dL (3.0 mmol/L)), respectively.

Environmental chemical exposure was also utilized to predict 2-h plasma glucose after OGTT (2-h PG after OGTT), blood insulin, diabetes mellitus, and FPG by LASSO regression and RF [130]. The LASSO regression predicted diabetes with an AUC of 0.80, while the linear model forecasted the glucose level.

A smartphone app (Diabits) was developed to assist patients with diabetes in managing and monitoring blood glucose levels [131]. To construct an applicable model, a combination of gradient-boosted decision trees (GBT) and SVM was used. A 30-min Diabits smartphone app forecasting assessed using Parkes Error Grid was computed to be 86.89% clinically accurate (zone A) and 99.56% clinically admissible (zones A and B), while 60-min predictions were 70.56% clinically accurate and 97.49% clinically admissible. It was determined that under free-living situations, several popular blood glucose control metrics ameliorated with the elevated frequency of app use. The 30-min predictions of the base Diabits model had a root mean square error of 18.68 mg/dL. These results hopefully showed that Diabits could accurately predict future glycemic fluctuations and help patients with diabetes to hold their blood glucose in the reference span.

A gradient-boosted tree algorithm was developed to forecast the response of the ICU patients concerning glycemic control as a significant parameter of critical care [132]. To provide convenient data for machine learning analysis, irregular multivariate time series data regarding the in-patient medical history and glycemic control comprising nutrition, previous blood glucose, and insulin dosing as electronic medical records (EMRs) were considered. Clarke error grid analysis showed that 97% of forecasting would be clinically admissible. The proposed model revealed a high degree of accuracy in predicting blood glucose in the range of 70–200 mg/dL. The mean absolute percentage error for the overall and surgical patients was determined as 16.5% and 15.1% for 2-hour predictions of serum blood glucose, respectively.

Several factors cause difficulty in glycemic control. The feed-forward artificial neural network’s predictive models were designed employing two hidden layers with 15 and 10 processing elements in each layer, respectively [133]. A collection of CGM data and other accompanying values from those who had been admitted to the ICU were used as input features information. The model was developed to forecast a complete path of glucose amounts up to 135 beforehand time. The mean absolute difference percent error was found as 15.9% respecting the serum blood glucose values and 10.6% regarding the interstitial glucose level. The Clarck EGA of model predictions than the reference CGM and blood glucose determinations disclosed that more than 99% of model predictions could be clinically accepted and would not be resulted in wrong insulin treatment or therapy recommendations.

Patients with T1DM should receive insulin to avoid the long-term consequence of hyperglycemia and they also should be cautious to take the proper amount of insulin and prevent hypoglycemia [134]. Therefore, the patients should obey a “regimen” to specify the injected value of insulin at each time. It becomes possible when the future of blood glucose amounts would be predicted from the current features. Twelve various supervised machine learners, based on seven learning algorithms including SVR, Wavelet Neural Networks (WNN), K-NN, Random Forest Regression (RFR), Gaussian Process Regression (GPR), ANN, and Ridge Regression (RR) were constructed. In addition, five more complex learners, by employing model stacking and also considering weighted ensembles of GPRs were tested. The results showed that the diabetes diary data were probably insufficient for generating accurate BG prediction models. It means that more information is required to construct accurate BG prediction models over hours.

The IoT devices and new emerging biosensors facilitate the continuous gathering of glucose amounts [135]. Using suitable ML algorithms, glucose assessment could be modeled to predict this variable. Furthermore, glycemia dynamics need that the model would be user-centric and the handles risk of lack of accessibility to the Internet and developing on-the-fly forecasting. To develop this process, some univariate algorithms have been applied in a Raspberry Pi and a smartphone, considering only previous glycemia data to predict glucose levels. The outcomes revealed a 15-min horizon with an RMSE of 11.65 mg/dL in only 16.15 s would be possible in a smartphone, using a random forest algorithm and 10-min sampling of the previous 6 h of data. With the Raspberry Pi, the computational effort was improved to 34.89 s employing SVM (RMSE of 19.90 mg/dL).

The assessment and modeling of glucose oscillations help forecast the time to inject insulin in T1DM subjects. To this end, Tesseratus hybrid model was developed to predict the glucose oscillation for up to 4 h during the daytime and for up to 8 h during the night period [136]. This model is based on both compartment models and data-driven algorithms like machine learning. It has two kinds of agents: reactive agents and intelligent agents. The reactive agents are accountable for gathering data and feeding intelligent agents with the accumulated data or for monitoring data, errors, and ODE parameters. Intelligent agents are responsible for utilizing data to forecast glucose oscillation and comprise the recommender agent, the predictor agent, the ML agent, and the math agent. It was claimed that Tesseratus could be a reference for the classification of a glucose prediction model which helps decrease long-term complications in T1DM individuals.

Several non-ensemble benchmark models including LR, vanilla LSTM, and Bidirectional LSTM (BiLSTM) as well as their contributions as base-learners for constructing the ensemble models were used to predict BG levels in 30 and 60 min [137]. The univariate time series predicting, multivariate time series predicting, and two-dimensional data analysis were used to fuse the outputs of the base learners. The performance order of models was as follows: ensemble models, non-ensemble models, and naive baseline model.

A weighted ensemble of RF, XGB, DT, LGB, and NB was used to early predict diabetes [138]. Grid search hyperparameter optimization was utilized to tune the hyperparameters of models. ANOVA test showed that the performance of diabetes prediction was remarkably amended when the weighted ensemble of DT + RF + XGB + LGB was executed (AUC: 0.832). However, the RF-based feature selection approach generated the best outcome for early diabetes forecasting.

The accurate prediction of BG level is still a challenge for diabetes management. Several factors such as diet, activities, stress, and personal physiological characteristics, affect the BG level, so the precise forecasting of BG is an open problem in diabetes management. A personalized model based on a CNN with a fine-tuning strategy was developed for accurate forecasting of BG on a dataset containing T1DM, T2DM, and GDM data [139]. Only CGM data points were used as input during the preprocessing and future BG amounts of 4 various prediction horizons (PHs, 15, 30, 45, and 60 min) were used as output. Then, a CNN and a multi-output random forest regressor employing a hold-out method for each group was trained. This model improved the performance of the general CNN in most cases. In addition, it was found that the BG level at the time of forecasting was related to the future BG level trend.

One of the beneficial preventive actions to manage diabetes is forecasting forthcoming levels of blood glucose concentrations. An RNN based on the LSTM model was developed for predicting upcoming blood glucose levels in T1DM subjects and then integrated with multiple insulin and carbohydrate absorption curves in order to optimize the prediction outcomes [140]. The accuracy level of around 0.510 mmol/L (9.2 mg/dl) was obtained.

Despite a large number of CGM data that provide the required data for developing deep learning algorithms for personalized BG forecasting in T1DM, uncertain forecasting confidence and limited training data for new T1DM individuals are challenging. Therefore, a Fast-adaptive and Confident Neural Network (FCNN) model was developed to overcome these clinical problems [141]. An attention-based recurrent neural network was employed to learn representations from CGM data and forward a weighted sum of hidden states to an output layer, aiming to calculate personalized BG forecasting. The model-agnostic meta-learning was used to provide fast adaptation for a new T1DM patient with limited training data. The FCNN was successful in predicting BG levels in T1DM and reached RMSE of 18.64 ± 2.60 mg/dL and 31.07 ± 3.62 mg/dL for 30 and 60-min prediction horizons, respectively. In another work, the forecasting glycemic levels of pediatric T1DM patients were investigated by CNN and LSTM-RNN models on insulin, glucose, and meal data [142]. The models were then executed on two edge-computing boards to assess the possibility of an edge system for glucose prediction in terms of forecasting accuracy and inference time. The LSTM model achieved the best clinical and numeric accuracy when tested in one format, whereas the CNN achieved the best clinical accuracy in another format. The performance and inference time were also acceptable for a real-time application. In another study, LSTM-RNNs were used to dynamically predict the next-day glucose levels in T2DM patients based on their daily mobile health lifestyle data on weight, physical activity, diet, and previous glucose levels [143]. A transfer learning strategy was also developed to cope with data scarcity and ameliorate forecasting accuracy for each patient. The suggested model showed remarkable accuracy in forecasting the next day’s glucose level based on the Clark Error Grid and the ± 10% range of the real values.

The prediction models including a non-linear autoregressive (NAR) neural network and LSTM were also trained on glucose signals of a heterogeneous and large cohort of patients and afterward, used to derive future glucose-level amounts in the new patients [144]. The prediction accuracy of NAR was good for prediction horizon within 30 min, however, the LSTM model yielded high performance both for short- and long-term glucose-level inference and was outperformed on feed-forward neural networks (FNNs), autoregressive (AR) models, and RNN. Therefore, it was concluded that LSTM is the premier method for systems with a very long-term predicting window.

In order to evaluate the effect of the step of feature selection (FS) in improving the accuracy of the predicted glycemia in T1DM subjects, six FS algorithms including LR, RF, Multi-Layer Perceptron (MLP), Instance-Based K-nearest neighbor (IBk), Relief Attribute (Rlf), and PCA beside four predictive algorithms (RF, LR, SVM, and GP) were applied to a biomedical features dataset [145]. The outcomes showed that RF as both FS technique and predictive algorithm causes the best RMSE (18.54 mg/dL) throughout the 12 considered predictive horizons (up to 60 min in stages of 5 min). In addition, applying SVM as a forecasting algorithm led to the best accuracy when the average of the six FS algorithms was applied (RMSE = 20.58 mg/dL).

The time delay in the CGM systems may lead to a considerable change between the actual BG and the CGM levels. To dominate this obstacle, an artificial neural network regression method was applied to forecast CGM amounts in T1DM patients with a lead-time of 15 min [146]. The external validation yielded an RMSE of 99.9–100%. In another work, a multilayer deep neural framework with a combination of LSTM with the gated recurrent unit (LS-GRUNet) was constructed to forecast the future glucose level in T1DM patients based on meal information and glucose levels for a prediction horizon of various times [147]. The RSMEs were 5.27 mg/dL and 14.85 mg/dL for 15 and 30 min prediction horizons which were comparable to existing methods in the literature.

The IoMT is described as the junction of multiple medical devices in healthcare systems. It supplies remarkable advantages in clinical applications when combined with AI technologies. A deep learning algorithm of attention-based lightweight RNN was proposed to be executed in an IoMT-enabled wearable device employing a system on a chip (SoC) for Bluetooth low energy (BLE) connectivity and edge computing [148]. An evidential regression was used to calculate model uncertainty and ameliorate the detection of impending hypoglycemia. After receiving the measurements from CGM, the wearable device carry out real-time model inference to acquire the BG forecasting and hypoglycemia warning for decision support. The embedded model was evaluated on the data from the T1DM subjects. The proposed model reached the premier performance of RMSE and acquired the best accuracy for hypoglycemia detection when compared with ARIMA, TCN, CRNN, LSTM, Bi-LSTM, and SVR approaches.

An LSTM network with one LSTM layer, one bi-directional LSTM layer along with several fully connected layers was applied to forecast BG concentration [149]. The LSTM network was first trained with both real T1DM patient and in silico data to develop a “global model”. Then, the model was trained and tested with multiple real datasets comprising more than 5-day CGM data. The model was fast and outperformed the baseline algorithms SVR and ARIMA with reduced RMSE and time lag, while the correlation coefficient and Fit were increased.

A deep learning method was developed based on function approximation on data-defined manifolds, employing diffusion polynomials [150]. The BG levels of various patients were taken at 5-min intervals with the CGM device to generate time series data. To quantify the clinical accuracy of the desired predictors, the Prediction Error-Grid Analysis (PRED-EGA) was applied. This assessment methodology records reference BG estimates along with the BG approximates forecasted for similar moments. The PRED-EGA reports the percentage of accurate, benign, and erroneous predictions in the hypoglycemic, euglycemic, and hyperglycemic ranges, individually. This stratification is important because the prediction errors in the different ranges lead to various consequences. In the first layer, the training patient was set by randomly selecting the patients. Then, a short-term prediction of the BG level after 5 min was performed by applying the linear predictor approach. According to these 5-min predictions, the measurements were clustered in three clusters. This step was carried out to collect more information concerning the training set. Afterward, the best one of the three predictions was determined. In the last training layer, the final output was generated based on which predictions give the best placement in the PRED-EGA grid. Eventually, to assess the performance of the final output, the actual reference values were used to place it in the PRED-EGA grid.

### Detection of blood glucose

To measure blood glucose concentration, a mathematical model based on energy metabolism conservation was proposed [151]. To this end, a multisensor integrated diagnosis probe put on the wrist was developed. Moreover, back-propagation neural network (BPNN) and multivariate polynomial regression were utilized to construct a regression model to measure BG concentrations. The Levenberg–Marquardt back-propagation algorithm was applied for training the model and tansig sigmoid functions were employed for the hidden layer. Further experiments showed the possibility of the proposed method in the detection of noninvasive BG concentration based on the conservation of energy metabolism. In addition, about 98.4% of the predicted BG amounts were inside area A of the Clarke error grid.

The Fourier-transform infrared spectra data of saliva was modeled by SVM, ANN, and LR to reach the following purposes: characterization of diabetic patients in uncontrolled and controlled based on their recorded pre-prandial HbA1C amounts, characterization of diabetic cases according to their pre-prandial glucose amounts obtained at the time of taking the saliva sample, and assessment of a specific glucose amount [152]. The outcomes revealed that the abovementioned examinations are possible through ANN using regression models.

A Complementary Ensemble Empirical Mode Decomposition(CEEMD) and Least Squares Support Vector Machine (LSSVM) were proposed to predict blood glucose concentration [153]. First, CEEMD was applied to transform the sequence of blood glucose concentration into a collection of intrinsic mode functions (IMFs) for decreasing the effect of nonstationary signals and also reducing the randomness of the prediction efficacy. Then, the LSSVM model was constructed for each mode IMF. After that, the comprehensive learning particle swarm optimization (CLPSO) algorithm was employed to optimize the kernel parameters of LSSVM. Eventually, the prediction outcomes of all IMFs were superimposed to obtain the blood glucose concentration prediction amount. The experimental results demonstrated the model has superior prediction accuracy in the short-term blood glucose concentration amounts.

Restricting the levels of blood glucose to be under the euglycaemic range could decrease the occurrence of diabetes-related consequences and amend people’s quality of life suffering from T1DM [154]. To forecast the sudden alterations in blood glucose amounts that are generated during physical activity, a Jump Neural Network model was developed. For this aim, three learning configurations including online training, offline training, and online training with reinforcement were evolved. All configurations were examined on six T1DM individuals who held regular PAs (three anaerobic and three aerobic) with controlled CGM. The online learning configurations carried out much better than the offline one on all days but not on the only CGM accompanied by the PA. The outcomes did not legitimize the elevated computational burden because the betterment was not remarkable. The RMSE for those who performed aerobic and anaerobic exercises were found as 26.3 and 20.8, respectively. The prognosis of T2DM could be accomplished through the control of blood glucose [155].

To predict glycemic, an Elastic Network (EN) for addressing the variable collinearity was combined with RF, SVM, and Back-Propagation Artificial Neural Network (BP-ANN) algorithms. In addition, a stepwise LR was carried out for comparing ML models. Basic information, diabetes-related data, and biochemical indices were used as variables. The multivariable analysis revealed that exercising, atherosclerotic cardiovascular disease history, hypertension history, and total cholesterol were protective factors in the control of glycosylated hemoglobin (HbA1c), while the family history, insulin dose, central adiposity, complications, T2DM duration, hypertension, and blood pressure were the risk factors for the increased HbA1c. After the dimensional reduction by EN, the AUC of SVM, RF, and BP were determined as 0.72, 0.75, and 0.72, respectively. Moreover, the EN and machine learning models had superior accuracy and sensitivity than the logistic regression models. The EN and ML algorithms could be considered the alternative to the traditional logistic model, to construct the predictive methods of blood glucose control in T2DM patients.

A personalized glucose monitoring system (PGMS) comprises both non-invasive and invasive sensors on a solitary device [156]. In one study, blood glucose data was used for training the ML models. Then, the paired data and corresponding errors were divided into 6 various clusters based on blood glucose ranges. Each cluster was trained to construct the inimitable error prediction model employing an AdaBoost approach. These error prediction models undergo personalized calibration according to the patient’s features. When the errors in forecasted amounts were within the admissible error range, the device got personalized for a patient to determine the blood glucose non-invasively. The performance analysis showed that the MARD was decreased to around 7.3% for the predicted amounts in comparison with 25.4% for the evaluated non-invasive glucose amounts.

To assess several variables extracted from lifestyle and medical self-monitoring data, RReliefF and RF approaches were initially used to rank the candidate variable set [157]. Then, a forward selection method was pursued to construct a glucose predictive model. In which, the related variables were consecutively surcharged to it in a subtractive order of significance. Predictions were carried out utilizing Gaussian or support vector regression processes. The glucose profile in addition to the time of the day and concentration of plasma insulin were highly ranked, while the efficacy of physical activity and food intake remarkably differed among cases. The prediction horizon (min)/RMSE (mg/dl) were obtained as 30/5.7 ± 1.5, 30/5.9 ± 1.4, 30/5.6 ± 1.7, and 30/5.9 ± 1.6 for SVR-RF, SVR-RRF, GP-RF, and GP-RRF, respectively.

An SVM binary classifier was developed with the purpose of determining if a CGM data stream pertains to an individual contributor [158]. To produce the variable vector employed for classification, the standard glycemic metrics were chosen and assessed at different time periods of the day (24 h, day, night, breakfast, lunch, and dinner). A recursive feature selection approach was used to choose the minimum subset of variables. A window length of 15 days was identified as the optimum interval for accuracy (86.8% ± 12.8%). The method was suggested as a digital CGM “fingerprint” or for detecting glycemic alterations within a subject.

### Insulin resistance predicting models

Insulin resistance (IR) is a situation in which cells in the liver, fat, and muscles have weak responses to insulin and are unable to utilize glucose from the blood for consuming energy. Insulin resistance can be used for the early detection of T2DM [159]. To predict insulin resistance using the ML approach, the potential impact of glucose, obesity, lipid metabolism, kidney operation, liver function, environmental factors, and genetics were considered to construct the model. LR, XGBoost, RF, and ANN algorithms produced the optimal prediction model for insulin resistance. XGBoost and LR created the greatest AUC (0.86) of the prediction models by employing 99 variables, while the RF produced a model with an AUC of 0.82. The models revealed that liver function, pulse, and seasonal variation, as well as metabolic syndrome components, should be noticed to predict insulin resistance in Koreans aged over 40.

An unsupervised machine learning method was utilized to assess the homeostatic model assessment-insulin resistance (HOMA-IR) cut-off to find individuals at risk of IR based on clinical data [160]. First, HOMA-IR-correlated features were determined by using a clustering algorithm, and two clusters with the lowest overlap in their HOMA-IR amounts were retrieved. These clusters were the samples of insulin-sensitive individuals and subjects who were at risk of IR. A total of 14 variables were finally selected: plasma leptin, two-hour postprandial glucose concentration, waist circumference, body mass index, total cholesterol, very-low-density lipoprotein, percentage of fat, alanine transaminase, TG, HbA1c, 24-hour systolic and diastolic blood pressure, free thyroxine, and human growth hormone. The cut-off amount of 1.62 ± 0.06 was found from the intersections of the Gaussian functions and then modeling the HOMA-IR distributions of these populations. Such an approach may determine high-risk subjects at an early stage, which then hamper or delay the initiation of T2DM.

Triglyceride-glucose (TyG) index has been presented as a beneficial element for determining insulin resistance and for the preliminary identification of cases at T2DM risk [161]. Based on this concept, a multiple instance learning boosting algorithm (MIL-Boost) was proposed to construct a predictive model for the early forecasting of worsening IR considering the TyG index. It was developed regarding the information of the past EHRs and also it was capable of extracting hidden patterns, even not the direct measurements of glucose and triglycerides. The suggested model was efficient to predict IR (Recall: 0.70–0.83).

For determining insulin resistance, specific blood tests are required. While the TyG index could provide a substitution evaluation from routine EHR data [162]. The ensemble regression forest combined with the data imputation strategies, namely, TyG-er was developed. The results diagnosed the non-conventional clinical factors like protein profile, leukocytes, uricemia, and gamma-glutamyltransferase. In addition, they could provide a novel intuition to the foremost combination of the clinical factors for discovering early glucose tolerance deterioration. The robustness of the elicited clinical factors was affirmed by the high settlement (0.664–0.911 of Lin’s correlation coefficient) of the TyG-er method among various experimental techniques. The combination of SVM-Gauss with the K-NN data imputation demonstrated the best predictive power. However, the TyG-er method was identified to be the foremost contender and it was suggested a higher level of interpretability in comparison with the SVM-Gauss approach.

To develop the ML-based methods for estimating insulin resistance in children with T1DM, both Multivariate Adaptive Regression Splines (MARSplines) and Artificial Neural Networks (ANN) were employed [163]. A hyperinsulinemic hyperglycemic clamp study was carried out to assess the Glucose Disposal Rate (GDR). The outcomes were compared with the predictive models based on HbA1c, TG, and waist circumference levels. The reference model demonstrated a moderate performance with a median absolute percentage error of 49.1%. Predictions of the MARSplines model were remarkably more accurate versus the reference model (median error 3.6%). On the other hand, the ANN predictions were displayed significantly in the lower error versus the MARSplines and also the reference model.

### Determination of the start and effect of treatment

Canagliflozin is an insulin-independent glucose-lowering compound that is used for diabetes patients due to its convenient influence on the renal and cardiovascular [164]. But, the clinical trials disclosed that there was an elevated risk of lower extremity amputations (LEA) accompanied by canagliflozin. To predict LEA, several ML algorithms were used. The LASSO method could produce the best prediction (C-statistic of 0.81) among others. For this reason, it was proposed that the identified risk score could help to obtain the best treatment decisions.

An empirical standard formula (SF) was frequently applied for checking the daily insulin injections and mealtime insulin boluses (MIB) to offset the deficiency of endogenous insulin generation because of β-cells destruction in T1DM [165]. However, SF may result in over/underestimations and cause serious hyper/hypoglycemic episodes during or after the meal. Therefore, GBT and RF nonlinear algorithms were utilized to overcome this challenge. The preprandial BG, glucose rate-of-change, and meal amounts were considered variables. The evaluations were carried out regarding accuracy in the assessment of the optimal glycemic and bolus control. The models with the remarkable improvements in glycemic management versus the linear approach showed decreasing in the time spent in hypoglycemia from 32.49 to 25.20%- 27.57 for GBT and RF, respectively. The result disclosed that the nonlinear machine learning approaches could ameliorate the approximation of insulin bolus in T1DM treatment.

Also, several models based on LASSO and multiple linear regression (MLR) were evaluated to develop the computation of MIB using the CGM data [166]. LASSO regression with an extended feature set including Quadratic terms (LASSO-Q) produced better results than the existing techniques. Moreover, LASSO-Q diminished the fault in assessing the optimal bolus and hypoglycemia occurrence. MIB dosage with the suggested LASSO-Q model could decrease the risk of harmful events in T1DM therapy. In another work, a novel method was developed based on neural networks, to personalize and optimize the bolus computation utilizing the information of continuous glucose monitoring and available parameters of patients. The neural network method led to a decrease in blood glucose risk index value equal to 0.37 versus SF [167].

It has been reported that T1DM patients need long-term exogenous insulin treatments to adjust their blood glucose levels [168]. Therefore, the automatic recommendation of personalized insulin dosage levels could be beneficial. To this end, a model-free data-driven RL approach, Q-learning, was developed to recommend insulin doses. The variables included engagement in physical activity, body mass index, alcohol usage, and glycated hemoglobin levels were considered. In this method, the RL agent found various patient states by tracking the patient’s replies when the case was subjected to varying insulin doses. Based on the outcome of treatment at a special time stage, the RL agent gave a numeric negative or positive reward. The reward was computed as a function of the discrepancy between the real blood glucose level obtained in response to the targeted HbA1c level and insulin dose. The interval of RL agent–recommended insulin dosage comprised the true dose that was endorsed by the doctors in 88% of the test patients. The method showed that an RL algorithm could be employed for recommending personalized insulin doses to achieve sufficient glycemic control in T1DM patients.

To evaluate the treatment results of T2DM and to recognize the characteristics of patients associated with the achievement of a target HbA1c of ≤ 7%, several ML methods were applied [169]. The data was the effect of a clinical trial assessing the single-pill combination of the sodium-glucose cotransporter-2/dipeptidyl peptidase-4 (SGLT2/DPP-4) inhibitor empagliflozin/linagliptin with linagliptin or empagliflozin monotherapies to identify novel predictors of remedy success, defined as HbA1c decrease. For the first time, descriptive analysis was applied to evaluate univariate associations between each baseline characteristic and HbA1c target categories. Then, RF and the classification tree algorithms were used in order to appraise and also to forecast the target groups based on the patient features from the baseline. This procedure could be done without any previous selection. In the descriptive analysis, FPG and lower mean baseline HbA1c were both correlated with the obtained HbA1c target. The ML analysis found FPG and HbA1c as powerful predictors for glycemic control. The covariates which included waist circumference, body weight, blood pressure, or other features did not contribute to the results. In the validation set, trivial progress in the prediction accuracy was acquired for the random forest model versus the classification tree model: 82% versus 80% for the empagliflozin, 81% versus 79% for the empagliflozin/linagliptin single-pill combination, and 78% versus 77% for the linagliptin.

Metformin is the common first-line drug for the treatment of prediabetes and T2DM [170]. However, it fails in the treatment of a third of cases. A machine learning model was developed to predict the blood glucose control in patients who have consumed metformin after one year of therapy. Predictors were derived from four main features: baseline HbA1c level, comorbidities, demographic variables, and baseline metformin dosage. A total of 20 base models containing a broad diversity of underlying methods including RL both with and without regularization and/or stepwise feature selection, tree-based models, SVM, multivariate adaptive regression splines, and flexible discriminants were constructed. The ensemble models comprised a non-linear stack of all base models, a linear stack of all base models, and a linear stack of a subset of maximally diverse models. The basic approach underlying stacking was used to train a pool of base classifiers on a training set. Then, it was utilized for training another classifier named a combiner on the predictions of the base classifiers. Various ML models were trained by employing variables existent at the time of metformin initiation for predicting the attainment and also for keeping HbA1c < 7.0% after one year of treatment. AUC performances were determined from 0.58 to 0.75. The most important features were the baseline HbA1c, the attendance of diabetes with complications, and the starting metformin dosage.

One of the main duties of precision medicine is developing Individualized Treatment Rules (ITRs) for patients with heterogeneous responses to the various treatments [171]. It led to finding and employing potential biomarkers beneficial to rectify an ITR. For this aim, the Net Benefit Index (NBI) was developed to quantify a contrast between the resulting loss and gain of therapy when a biomarker entered ITR to reassign patients to therapies. Moreover, a weighted SVM was used to find the optimal treatment group labels. Through the proposed index, the baseline fasting insulin was found as a significant biomarker. Applying this protocol resulted in progress over an existing ITR and decreasing in FPG over 52 weeks.

GDM is a major challenge that can create morbidity in women and newborns [172]. However, monitoring the woman’s blood glucose and considering the risk factors could help in making decisions for the commencement of treatment by metformin or insulin. Mobile Health (mHealth) solutions provided real-time follow-up and authorized timely therapy and management. By applying a logistic machine learning algorithm, the timing of the pharmacological treatment beginning was accurately predicted with Insulin therapy ( AUC = 0.8). It was commonly recommended for patients with T2DM once per day.

Calculating the missing dose is crucial as the daily insulin dose can be forgotten by the patients [173]. Therefore, determining a missing injection based on CGM data from the same day could be beneficial because the dose must be taken within 8 h of the next injection. To detect daily adherence, deep learning methods based on convolutional neural networks were surveyed and compared with ordinary feature-engineered ML classification models. Six different models obtained from the automatically learned features and also the expert-dependent were examined. Three classification models based on the expert-engineered features acquired mean accuracies of around 78%. One of these classification methods relied merely on learned variables and got a mean accuracy of 79.7%. The two other classification techniques including fusing expert-engineered and learned variables achieved mean accuracies of 79.8% and 79.7%, respectively. The accuracy of adherence detection was improved when more CGM data became accessible on the day of classification.

Blood glycemic control is essential to minimize the side effects of diabetes [174]. Two contrary treatment methods have been presented: formulaic and closed-loop approaches. In formulaic approaches, insulin care is computed by parameter-based calculation (i.e., insulin-to-carb ratio, correction factor, and absorption duration), which are determined based on the history of examined blood glucose levels. Alternatively, closed-loop approaches examine the glycemic level through the sensors and supply the insulin boluses according to the data of the sensor. The abovementioned systems are reactive leading to the remarkable fluctuations of glucose amounts, which ultimately resulted in hyperglycemia. Therefore, the reaction of patients to insulin therapy was modeled by Markov Decision Process (MDP) which allowed the system to identify an unparalleled, individualized and dynamically updated insulin care policy. The solution to MDP was identified by reinforcement learning. This approach could hamper hypoglycemia and also could lead to decreasing both glycemic fluctuations and high glucose duration. In addition, this method could allow the care team to update the patient model and better support.

In order to predict and avoid the occurrence of NH in individuals with T1DM under multiple-dose insulin (MDI) therapy, various data sources, ML algorithms, and optimization metrics were assessed [175]. ML methods including multinomial NB, ANN, SVM, AdaBoost, linear discriminant analysis (LDA), and LSTM were examined. The outcomes revealed the foremost results for the SVM algorithm. Moreover, the population and personalized models were designed and the impact of physical activity was evaluated. The results showed that 30 g of rescue carbohydrates is the optimal amount for avoiding NH. Therefore, the positive effect of BG predictions to make accurate decisions regarding insulin therapy and day-to-day lives was concluded.

A step-wise approach was proposed to choose drug combinations for compensating carbohydrate metabolism for T2DM patients [176]. The main carbohydrate metabolism indicator – glycated hemoglobin, arterial blood pressure, and the lipid profile indicator – low-density lipoprotein cholesterol were used as indicators for the prediction. The approach comprised the following stages: (i) using machine learning regressors including RF, CatBoost, and XGB for forecasting the future impact of treatment; (ii) using a Bayesian network for the personalized computation of pharmacological categories that are needed for the patient; (iii) employing a modified genetic algorithm for detecting the best combination of drugs for each patient. The method was assessed and validated using virtual implementation and by comparing the predicted outcomes with the real treatments.

### Risk assessment of diabetes

Many individuals who have prediabetes and even T2DM or T1DM do not have any signs at first. It causes to increase in the risk of developing further diseases. Moreover, The American Diabetes Association (ADA) declares that the increase in risk factors can elevate the probability of appearing diabetes. Therefore, finding the risk factors and assessing the risk of developing diabetes can help to decrease further complications and also timely treatment.

The effect and usefulness of lipid and HbA1c variability for risk assessment in diabetes mellitus were evaluated by regularized and weighted Random Survival Forests (RSF) models. These techniques that were introduced as a class of machine learning methods were examined for survival analysis [177]. The outcomes revealed that the increase in the variability in lipid and HbA1c parameters were accompanied by the high risk of both diabetic difficulties and all-cause mortality. It was also concluded that the dependency between baseline Neutrophil- Lymphocyte Ratio (NLR), hypoglycemic frequency, and both lipid and HbA1c variability provided a major function for inflammation in mediating harmful results in diabetes. However, it is required to be investigated in future studies.

In another study, Deep Neural Survival learning models (DeepSurv) and RSF were employed to construct reliable methods considering variables including the variability values of HbA1c and fasting glucose, comorbidities, drug prescription details, and nutritional inflammatory indices [178]. The results showed remarkable predictive accuracy in comparison with Cox regression-based techniques.

To identify susceptible individuals with the risk of T2DM or prediabetes who had normal glucose regulation, an RF classifier was employed. This classifier could predict the low and high-risk features for glucose disorders at follow-ups between 10 and 20 years [179]. Afterward, SHAP TreeExplainer was utilized to interpret the results of the RF classifier. Age, BMI, systolic and diastolic blood pressure, waist-hip ratio, and diabetes heredity were identified as the important features so that their high values could lead to an increased risk of T2DM. Most features also had a role in the metabolic control of diabetes, so they could be employed in diabetes care to create personalized health care plans.

A risk assessment system was applied to predict the 3-year risk of occurrence of diabetes through a combination of clinical and demographic features [180]. For this reason, the XGBoost was applied to choose the reliable variables. BMI, FPG, and age were identified as the top three substantial elements.

An interpretable ML framework was applied to find the T2DM-related gut microbiome variables in the cross-sectional analyses of three Chinese cohorts [181]. To this end, a model based on a GBDT algorithm and LightGBM was developed to connect input features with T2DM. Moreover, SHAP was applied to untie the machine learning outcomes. The mean absolute value of the SHAP showed the average share of each feature to the overall model predictions. Therefore, features with an average absolute SHAP value higher than 0 were considered as chosen features. Afterward, a Microbiome Risk Score (MRS) was constructed with the determined features, and its association with glucose, demographic, dietary factors, and adiposity was evaluated. The MRS was positively correlated with future glucose increment and diversity of gut microbiota-derived blood metabolites. The distribution of body fat was identified to be a critical factor modulating the gut microbiome–T2DM relationship.

In one study, NB and LR approaches were utilized to build a risk prediction model for Large-For-Gestational-Age (LGA) infants using a large multi-center cohort [182]. Models were developed by integrating the demographic, clinical variables, and risks of hyperglycemia as potential predictors. In this case, hyperglycemia was evaluated in three parts: GDM subtype, IADPSG GDM yes/no, and OGTT z-score quintiles. Both methods led to the same approximation for the LGA risk, however, the AUC for the LR model was significantly higher (0.698 vs. 0.682). The utilization of the individual OGTT z-score quintiles caused statistically higher AUC than the others.

To specify the T2D patients who were at elevated risk of hypoglycemia, a screening tool based on ridge logistic regression, logistic regression with backward selection, LASSO LR, elastic net logistic regression, and RF was proposed [183]. The medication and demographic data or the ensemble containing medication, demographic, and clinical data were utilized as predictor features for training models. The LR with the least absolute shrinkage and selection operator was found as the best accurate model including only demographic and medication data (AUC of 0.71). The significant features were ‘insulin use’, followed by ‘sulfonylurea use’ and ‘insulin use duration’. The suggested model acquired the same performance as the model employing the additional clinical data. Six ML approaches, including Classification And Regression Tree (CART), RF, GBM, LR, SVM, and ANN were used to develop the risk assessment models for predicting the risk of T2DM in the rural Chinese population [184]. All these models showed a potent predictive performance, with AUCs ranging between 0.767 and 0.817 without laboratory data as well as 0.811 and 0.872 using laboratory findings and experimental data. Among them, the GBM model was the best. The variables including urine glucose, sweet flavor, pulse pressure, age, creatinine, heart rate, waist circumference, insulin, uric acid, and hypertension were the top-ten selected features. Through this study, sweet flavor and urinary indicators were found as novel variables.

Abdominal computed tomography (CT) is an imaging technique that could be used to evaluate the risk of T2DM for about one future year [185]. The role of five various kinds of EMR was considered in prediction including (1) demographics (2) desired visceral/subcutaneous fat volumes in the L2 region (3) pancreas volume (4) glucose lab tests and (5) abdominal body fat distribution. Next, a deep neural network was constructed to forecast the onset of T2DM with pancreas imaging slices. Finally, a merged framework was built to combine EMR information and CT imaging slices to refine the prediction which led to a 4.25% and 6.93% AUC increase for estimating T2DM compared with utilizing EMR or images.

In another study, to forecast the risk of hypoglycemia, four years of electronic health data including the laboratory and the point-of-care BG values were used. These findings were employed for determining the clinical and the biochemical remarkable hypoglycemic episodes, BG </=2.9 and </=3.9 mmol/L, respectively [186]. The administered medications, laboratory results, vital signs, demographics, and procedures that were performed during the hospital stay were used to inform the model. The predictive variables from the LR model contained weight, people undergoing procedures, oxygen saturation level, albumin levels, type of diabetes, and use of medications (insulin, metformin, and sulfonylurea). XGBoost model had the best performance (AUC = 0.96) and it outperformed the LR model (AUC = 0.75) for the risk assessment of clinically substantial hypoglycemia. Therefore, advanced machine learning models were introduced as the prior models rather than the logistic regression models in forecasting the risk of hypoglycemia in patients with diabetes.

To assess the risk of T2DM using biochemical biomarkers such as C-reactive protein, bilirubin, fasting glucose, HbAc, and cholesterol, the LR model was applied [187]. The best performance was obtained when all predictors were used and the only cases with linearly growing glucose levels were considered. The accuracy of the model was 73.85%, AUC was 0.81, the sensitivity was 90%, and specificity was 42%.

One of the first approaches for the risk assessment of nocturnal hypoglycemia is relying on the Low Blood Glucose Index (LBGI) that has been proposed for Accu-Chek® Connect as a hypoglycemia risk index [188]. A general method that was proposed by combining the NH predictors was built from various glucose control indices (GCI). The suggested model was constructed based on four GCI associated with hypoglycemia. The aggregation method that led to improving the performance included a positive predictive value of 80.2%, a specificity of 83.4%, a sensitivity of 77%, and a negative predictive value of 80.6%. The obtained amount was greater than the magnitude that conventionally was noticed as acceptable.

To determine the elevated risk of progressing T2DM among the prediabetic cases, a model of multiple laboratories and clinical variables was developed to forecast the enlargement of T2DM within two years [189]. A supervised ML algorithm was improved for determining at-risk cases among hypertensive and obese patients. Employing a time series analysis with the variables of every case, one linear regression line and also one slope per variable were calculated. Then, features were comprised in a K-NN classification model. The importance of features was evaluated by employing the random forest algorithm. The K-NN model accurately classified 96% of cases, with a positive predictive amount of 96%, a specificity of 78%, a sensitivity of 99%, and a negative predictive amount of 94%. The RF algorithm elected the homeostatic model assessment–estimated insulin levels, insulin resistance, and body mass index as the most significant factors. The performance of this model corresponding with K-NN had an accuracy of 99% with a specificity of 97% and a sensitivity of 99%. As a result, this combined prognostic model could determine hypertensive and obese patients at risk for progressing T2DM within 2 years.

Several ML algorithms including BN, ANN, classification and regression tree, CHAID, discriminate (D), quick unbiased efficient statistical tree (QUEST), and ensemble models were assessed to predict the poor glycemic control and risks of complications in nonadherent T2D [190]. The highest AUC of the testing set for diabetic peripheral neuropathy, diabetic nephropathy, the diabetic eye, diabetic angiopathy disease, and glycosylated hemoglobin A were 0.859 ± 0.050, 0.902 ± 0.040, 0.832 ± 0.086, 0.889 ± 0.059, and 0.825 ± 0.092, respectively. The results disclosed that both ML algorithms and univariate analysis resulted in a similar conclusion. The duration of unadjusted hypoglycemic treatment and the duration of T2DM were found to be the critical risk factors for diabetic complications. Also, the number of hypoglycemic drugs was identified as the critical risk factor for glycemic control of nonadherent T2DM.

In another study, a feature extraction procedure was utilized to find the elements for predicting the future hypoglycemia risk [191]. To this end, short-term (less than one hour), medium-term (1–4 h), and long-term (more than four hours) patterns from the CGM signal, as well as contextual, demographic, interaction, and nonlinear features were employed. In addition, two methods including LR and RF were applied. Feature selection for LR was carried out through the addition of a LASSO penalty. LASSO elevated an additional tuning parameter to the LR equation which defined a penalty for each variable involved in the model. In other words, a variable could be involved in the model when the amount of the modified loss function declines. The coefficient for an inconsequential variable was decreased toward zero and it could minimize its influence on the model. The optimal amount of the tuning parameter was specified iteratively. This process occurred by considering various penalty amounts and also by choosing a value that could minimize misclassification. Feature selection was carried out by employing the Variable Importance Plot (VIP), which obtained the average recovery in the class purity for splits involving a feature across all the ensemble trees. VIP was utilized to order the variables according to their misclassification influence. In this category, those parameters with unimportant impacts were discarded. This model predicted hypoglycemia with sensitivity and specificity of more than 91% and 90%, respectively, for 30- and 60-minute prediction horizons. The inclusion of carbohydrate and insulin data led to the improvement of performance for 60-minute, but not for 30-minute predictions. Moreover, the performance was superior for nocturnal hypoglycemia (~ 95% sensitivity).

Lifestyle affects diseases and mortality rates worldwide. Therefore, healthy weight, physical activity, and a healthy diet are important factors in prohibitive health care that could aid in decreasing the risk of developing T2DM [192]. To follow the factors impressing T2DM self-management and prohibition through lifestyle alterations, the E-App was developed. This app was capable of following the health data and dietary consumption, and also chasing walking, sitting, and running. Bluetooth movement data could be obtained from a pair of wearable insole devices that were employed to pursue blood glucose, carbohydrate intake, physical activity, and medication adherence. Two ML methods including DT and SVM models were employed to analyze the data which resulted in 86% accuracy.

Mobile health and self-management solutions simplify remote monitoring and contact without regard to location, time, or expense. In addition, each day’s meal can be registered and reviewed by the individuals. It authorized patients to do self-management and to follow their development.

The risk assessment LR and Xgboost models based on socio-demographics, lifestyle, and traditional risk factors were built to identify pre‐diabetes mellitus and diabetes mellitus. The SHAP approach was employed to assess the significance of the risk factors and to demonstrate the nonlinear relationship inside the Xgboost model. Moreover, the Boruta algorithm was utilized to choose substantial statistical risk factors by introducing shadow (randomized) variables. The results showed that BMI, waist‐to‐hip ratio, waist circumference, age, systolic blood pressure, smoking status, sleep duration, and vigorous recreational activity time were the remarkable risk factors of pre‐diabetes mellitus and diabetes mellitus [193].

### Dietary and insulin dose modifications

Although dietary modifications are decisive for managing T2DM, it is not successful for a large number of patients as an alternative to clinical treatments [194]. As a result, the clinical impacts of an algorithm-based Personalized Postprandial-Targeting (PPT) diet were used in the control of glycemic and metabolic health in patients with newly diagnosed T2DM. This diet method was compared with the usual recommended Mediterranean (MED) diet.

The PPT diet ameliorated CGM-based measures of glucose fluctuations, postprandial glucose responses, and daily time with glucose levels > 140 mg/dl versus a MED diet. The 6-month PPT intervention led to a remarkable development in several metabolic parameters, including fasting glucose, HbA1c, HOMA-IR, blood triglycerides, and daily time with glucose levels > 140 mg/dl.

An adaptive Basal-Bolus Algorithm (ABBA) was developed to present the personalized offer for the prandial insulin doses and the daily basal rate based on the glucose level of patients on the last day [195]. The data from both CGM and SMBG devices were accepted for use/adaption in the algorithm. The ABBA relied on RL and the variables containing one daily bedtime snack, three main meals, various uncertainties for insulin sensitivity, glucose measurement time, carbohydrate amount, and mealtime. The results demonstrated that artificial intelligence algorithms could present the optimization of personalized adaptive insulin. Also, these computational techniques could appear the glucose control regardless of the type of glucose monitoring devices.

The process of insulin injection to manage the blood glucose level is called fumigation. T1DM individuals have to inject insulin up to five times per day to balance their blood glucose levels. The calculation of convenient doses of insulin is often a challenge for patients. Therefore, DT, KNN, and ANN algorithms were used to follow the glucose levels and forecast the suitable amounts of insulin for T1DM patients [196]. The best result was obtained by the ANN model with an MSE of 5.79. This model was adapted to Raspberry Pi to measure the blood glucose amount and predict the appropriate insulin value.

A model-free reinforcement algorithm called the normalized advantage function (NAF) algorithm was used to regulate the BG level of T2DM patients through subcutaneous insulin injection [197]. The model-free aids to avoid inaccuracies and parameter uncertainty due to the mathematical models of the glucoregulatory system. The injected levels of insulin at each time frame were suggested by NAF to minimize the cost induced by hyper- and hypoglycemic events. NAF is capable of regulating and decreasing the BG fluctuations without meal declarations in comparison to standard optimized open-loop basal-bolus treatments. Considering the elimination of the insulin dynamic, an accurate and more realistic model was constructed than the formerly suggested models.

Hybrid closed-loop systems usually use simple control algorithms to choose the optimal insulin dose for T1DM patients. Online RL has been used as an efficient approach for further glucose control in these devices. In order to assess the performance of offline RL in these systems, the performance of batch-constrained deep Q-learning, conservative Q-learning, and twin-delayed deep deterministic policy gradient with behavioral cloning was compared with online RL [198]. The offline RL is used for developing clinically impressive dosing policies without the requirement for patient interaction. The offline RL could remarkably elevate time in the healthy BG range when trained on less than a tenth of the data needed by online RL methods. It was also capable of correcting false bolus and irregular meal timings.

### Diabetes management

Diabetes management needs consciousness. The factors that can influence the glucose blood and also the ways that can control it, should be recognized day-to-day. CGM could facilitate the prediction of the future glucose concentration for managing diabetes [199]. The glucose concentration amounts are impressed by insulin and meals as well as different metabolic and physiological variables including Acute Psychological Stress (APS) and physical activity. To survey the effects of APS and PA on the GC predictions, machine learning techniques including LDA, ensemble learning, GPR, KNN, SVM, DTs, and deep neural networks with LSTM were considered. The results revealed that the effect of PA on GC was much more considerable than APS. In addition, the PA effects were longer in duration as glucose decreased and long-term efficacies of alterations in insulin sensitivity led to a prolonged decline in CGM values. Under more stressful situations, the glycemic effects of APS were more substantial and the model would perform better. The MAE of one-hour-ahead GC forecasting with testing data reduced from 35.1 to 31.9 mg/dL with the admission of PA data, and it diminished from 16.9 to 14.2 mg/dL with the involvement of PA and APS data.

Three ML methods including RF, regularized regression, and GBT were employed to predict the amounts of the received insulin in two groups consisting of more than 6 units (“higher” insulin users) or 6 units (“low” insulin users) [200]. To combine these methods, SuperLearner algorithm was used to obtain higher performance. Moreover, the point-value Total Daily Dose (TDD) was forecasted in “higher” users, because there are broad variations in individuals’ responses to insulin. The outcomes showed that ML methods based on accessible electronic medical records could discern which inpatients needed TDD higher than 6 units. In addition, it could evaluate the individual doses more precisely than the standard guidelines. The method received an AUC of 0.85 for classifying the patients who needed > 6 units TDD and the mean absolute percent error with the dose prediction in the range of 136-329%. The regression model based on the weight ameliorated to 60%, and the full ensemble model progressed to 51%.

The control of T1DM relies on the suitable assessment of the insulin units to maintain the BG levels in the desired range [201]. This value is disparate for the patients depending on the calories they consumed and the degree of physical exercise they performed. The kinetics of body glucose is complex and different for each user. An RNN model based on LSTM cells could be employed to elevate the precision of blood glucose predictions according to the insulin absorption curves and the approximation of carbohydrate digestion for specified patients. The outcomes disclosed the capability of the suggested model to assess the absorption curves of insulin for normalized fast insulin curves that could have an uppermost amount of 1 unit. Moreover, the algorithm was capable of learning the complicated dependencies between the levels of blood sugar and estimated quantities for carbohydrate and blood insulin concentrations.

A predictive warning for the forthcoming hypoglycemic occurrence could help patients with T1DM to make a preventive decision to limit further consequences [202]. To construct such a prediction model with a low False Alert Rate (FAR) and proper generalizability to new individuals and time periods, RF and quantile regression forest were employed. Two diverse modeling methods were examined including classification-based and regression-based approaches. The classification-based method could straightly predict the occurrence of sustained hypoglycemia. However, the regression-based technique could forecast the glucose at several time points and also could provide a further deduction for sustained hypoglycemia. The sustained hypoglycemic occurrence was specified glucose amounts less than 70 mg/dL for at least 15 min. The model predicted sustained events with more than 97% specificity and sensitivity for both 30- and 60-minute prediction horizons. The FAR remained at less than 25%. The results confirmed that making alerts based on sustained events instead of all hypoglycemic occurrences could decrease the FAR and also could lead to the models with superior generalizability with the new individuals and period times.

One of the most concerning regarding T1DM management is the consumption of the correct amount of insulin for each meal that will correspond to the postprandial glycemic response (PPGR). In order to propose a prediction model for PPGR, the devised machine learning algorithm was applied [203]. The input data were the integration of glucose measurements, blood parameters, dietary habits, insulin dosages, exercise, gut microbiota, and anthropometrics. The model significantly was better with a correlation of R = 0.59 than a baseline model with R = 0.40 for observed PPGR. The model was also robust across various subpopulations. Feature attribution analysis disclosed that glucose levels at meal initiation, meal carbohydrate content, meal’s carbohydrate-to-fat ratio, and glucose trend 30 min prior to the meal were the most effective variables for the model.

An Electrocardiogram (ECG) has been surveyed to diagnose hyperglycemia, as it could influence the ECG signals. To do this, a 10-layer deep learning-based was developed [204]. The ECG data were first filtered by employing the Butterworth bandpass filter order 4 and a frequency range of 1 to 40 Hz. To find the cardiac cycles, the R-peaks were determined using the Pan–Tompkins that allowed a segment of individual heartbeats and further analyzing the cycle for the remaining waves. The remaining waves— P, Q, S, T—were then found with the aid of the NeuroKit library. Afterward, several experiments were carried out by examining various fiducial-based features that could provide better performance than employing the total cardiac cycle data as the input model.

A collection of 18 features composed of 9 fiducial distances were identified. QT interval was the time from the onset of the Q wave to the end of the T wave. The QT interval is influenced by both the people’s heart rate and glucose concentration, therefore, it is essential to reduce heart rate interference. In this case, the Framingham formula was used as the best correction. Finally, the outliers were removed and the data were normalized. The outcome revealed that the suggested algorithm was beneficial in diagnosing hyperglycemia with an 85.04% specificity, 87.57% sensitivity, and 94.53% AUC in a relative improvement of 53% against the previous models.

The safety in T1D management was assessed by four machine learning approaches including (1) SVM to forecast hypoglycemic incidence during postprandial periods, (2) grammatical assessment for the mid-term continuous forecasting of blood glucose levels, (3) data mining to profile diabetes management scenarios, (4) artificial neural networks to forestall hypoglycemic episodes overnight [205]. The aforementioned methods were used in various datasets for patient condition evaluation, continuous glucose level divination, prediction of nocturnal, and forecasting of postprandial hypoglycemic events. The combination of various models enhanced the feasibility of prognosticating factors.

Simultaneous personalized prediction models can make possible the assessment of an integrated and robust system for the prohibition of hypoglycemic events. This achievement could occur in both continuous subcutaneous-insulin infusion and Multiple Daily Injections (MDI) users. The proposed system remarkably decreased the number of hypoglycemia episodes. It led to providing more safety and higher assurance in decision-making for patients. Over 40% of people with T1DM should manage their glucose levels by MDI [206]. Wrong dosing (or “mistake in dosing”) could result in the incident of hyperglycemia (> 180 mg /dl) and hypoglycemia (< 70 mg/dl). A novel algorithm was developed that could weekly recommend the insulin dosage to T1D patients who used MDI treatment. The K-Nearest Neighbors-Decision Support System (KNN-DSS) algorithm obtained a settlement with the board-certified endocrinologists of 67.9% when it was confirmed on real-world human data. The analogy between the inter-physician-recommended regulations and the insulin pump treatment revealed a complete accord of 41.2% among endocrinologists. The ultimate result was in agreement with the previous values of inter-physician agreement (41–45%). The findings showed that the KNN-DSS could be implemented for the primary recognition of dangerous insulin regimens. Also, it could be applied to ameliorate glycemic outcomes and barricade life-threatening consequences in people with T1DM.

The precise assessments for the near future blood glucose levels have been momentous for the T1DM patients to respond on time and prevent hyper- or hypo-glycemic events [207]. Numerous models have been proposed based on regulating the glucose physiological-metabolic and machine learning. A hybrid model comprising the decomposing of a deep ML model to imitate the metabolic manner of physiological blood glucose approaches was also proposed to predict horizons of 30 to 60 min. The current and past BG evaluations, meal intake, and fast and slow-acting insulin injections were used as the variables. The differential equations for insulin and carbohydrate absorption in physiological models were modeled employing an RNN executed using LSTM cells. The results revealed RMSE of less than 5 mg/dL for the simulated patients by the AIDA diabetes software program and below 10 mg/dL for the real patients.

In T1DM, daily activity is considered an important factor in the calculation of insulin dose [208]. Bolus advisors have been presented to propose meal-related insulin doses based on carbohydrate consumption, pre-set insulin to insulin sensitivity factors, and carbohydrate levels. These parameters could be changed regarding regular activities. The main concepts with reporting and regulations of the usual occupation are based on self-reporting that is susceptible to inaccuracy. Furthermore, changing daily routines could be occurred inevitably through passing time. So, disremembering them in the bolus calculator could lead to substandard self-management. To overcome these limitations, time-series K-means clustering was employed to perform the significant separation of the patterns. It was thereupon applied to find the daily time periods and to recommend any required time alteration. The suggested model prepared a rapid, more precise, and individualized daily time setting. Also, it could supply more contextual outlooks to the glycemic pattern determination for both clinicians and patients.

In the daily handling of T1DM, understanding the true injected insulin dose at meal-time is pivotal to obtaining optimal glycemic control [209]. The CGM data accompanied by bolus insulin and carbohydrate intake classifying at meal-time and the post-prandial glycemic status could be utilized to determine the progress of the insulin treatment by decreasing or growing the corresponding meal bolus dose. To this end, an XBM algorithm was implemented to categorize the post-prandial glycemic status. The AUC of the suggested XGB algorithm was obtained as 0.84 and glycemic control in comparison with the baseline bolus calculator was improved.

Glycemic variability (GV) is a significant component of overall glycemic control for cases with diabetes mellitus [210]. To develop the Consensus Perceived Glycemic Variability (CPGV) metric that could evaluate diabetes mellitus control, SVR and multilayer perceptrons (MP) were used. The 250 24 h CGM plots were first rated in low, borderline, high, or extremely high GV, then their average was entered into the mentioned ML algorithms as input data. The SVR models estimated the unseen CGM plots better than the MP models and could obtain specificity, sensitivity, and accuracy of 74.1%, 97.0%, and 90.1%, respectively.

The prohibition of hypoglycemic events was important in the daily management of insulin-treated diabetes [211]. The usage of short-term prediction approaches for the subcutaneous glucose concentration could remarkably help toward this objective. An SVR method was used to predict hypoglycemia. This model forecasted the non- nocturnal (i.e., diurnal) ones over 30-min and 60-min horizons. Nocturnal hypoglycemic events were predicted with a sensitivity of 94% for both horizons and with time lags of 4.57 and 5.43 min. For the diurnal episodes, when physical activities were not considered, the sensitivity was 92% and 96% for a 30-min and 60-min horizon, respectively and time lags were less than 5 min. In the presence of such information, the diurnal sensitivity declined by 8% and 3%, respectively. Both diurnal and nocturnal predictions indicated a high (> 90%) precision.

Sometimes, infection occurrence could lead to hyperglycemia and repeated insulin injections in the T1DM subjects [212]. To create a personalized health model with the capability of forecasting the incidence of infection in people with T1DM, multiple boundaries and domain-based, density-based, reconstruction-based, and unsupervised models were constructed using insulin-to-carbohydrate ratio and blood glucose levels as input variables. The one-class classifiers achieved superior performance to diagnose the deviations from normal situations. It occurred due to the observation of infection incidences that could increase blood glucose levels in association with abnormal alterations in the insulin-to-carbohydrate ratio. Among the one-class classifiers, the domain and boundary-based approach generated a better description of the data. Moreover, support vector data description, nearest-neighbor, and self-organizing map needed significant training time.

## Future perspective

The overview of the published papers discloses that machine learning models can effectively improve the prediction and management of blood glucose and diabetes. However, they should be improved and surveyed in large datasets. A significant advantage of these models is that they can be used as an application on mobile or any other diabetes management device. This can help for ensuring the health of diabetic patients and also for preventing further complications. Moreover, finding the potential risk factors can help to assess healthy subjects to prevent getting diabetes. It is predicted that the future prognosis of diseases and therapy lines depends on developing powerful machine learning methods.

## Data Availability

Not applicable.
